# Developmental and Functional Control of Natural Killer Cells by Cytokines

**DOI:** 10.3389/fimmu.2017.00930

**Published:** 2017-08-04

**Authors:** Yang Wu, Zhigang Tian, Haiming Wei

**Affiliations:** ^1^Institute of Immunology and the CAS Key Laboratory of Innate Immunity and Chronic Disease, School of Life Sciences and Medical Center, University of Science and Technology of China, Hefei, China; ^2^Hefei National Laboratory for Physical Sciences at Microscale, University of Science and Technology of China, Hefei, China

**Keywords:** natural killer cells, cytokines, development, cytotoxicity, expansion

## Abstract

Natural killer (NK) cells are effective in combating infections and tumors and as such are tempting for adoptive transfer therapy. However, they are not homogeneous but can be divided into three main subsets, including cytotoxic, tolerant, and regulatory NK cells, with disparate phenotypes and functions in diverse tissues. The development and functions of such NK cells are controlled by various cytokines, such as fms-like tyrosine kinase 3 ligand (FL), kit ligand (KL), interleukin (IL)-3, IL-10, IL-12, IL-18, transforming growth factor-β, and common-γ chain family cytokines, which operate at different stages by regulating distinct signaling pathways. Nevertheless, the specific roles of each cytokine that regulates NK cell development or that shapes different NK cell functions remain unclear. In this review, we attempt to describe the characteristics of each cytokine and the existing protocols to expand NK cells using different combinations of cytokines and feeder cells. A comprehensive understanding of the role of cytokines in NK cell development and function will aid the generation of better efficacy for adoptive NK cell treatment.

## Introduction

**Figure 1:**
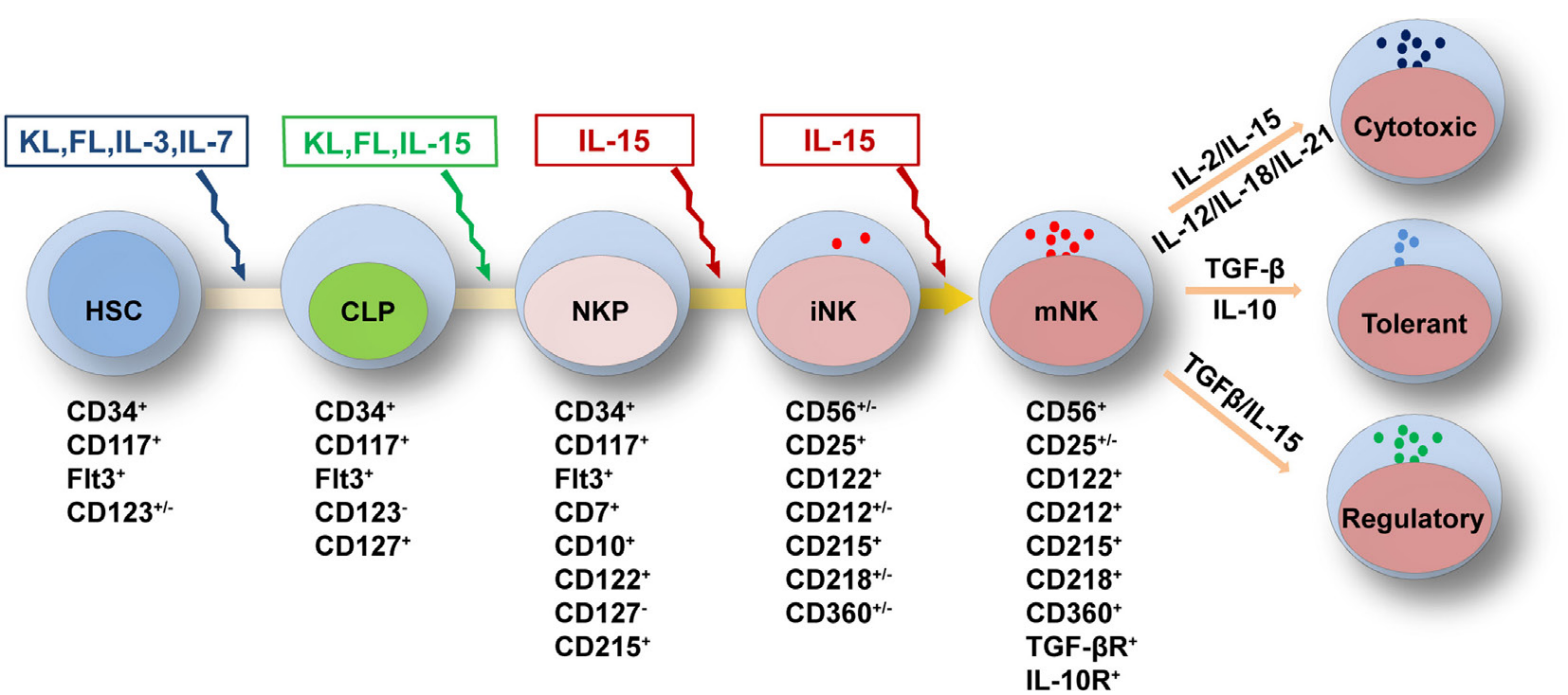
Cytokine requirements for natural killer (NK) cell development and function. NK cell development from HSCs is regulated by multiple cytokines in the fetal liver, bone marrow, and thymus. The sequential expression of receptors for different cytokines implies the functional maturation of NK cells. The proliferation and differentiation of HSCs requires FL, KL, IL-3, and IL-7, which interact with their respective receptors. The acquisition of CD122 expression is indicative of the commitment of NK cells. IL-15 is indispensable for NK cell differentiation from CLPs to mature NK cells. Mature NK cells are shaped by cytokine signals from the diverse tissue environments in which they reside. In the peripheral blood or spleen, the abundance of stimulatory cytokines, such as IL-2, IL-12, IL-15, IL-18, and IL-21, may maintain NK cells in a cytotoxic state to combat infections. Tolerant NK cells that reside in the liver, and regulatory NK cells that reside in the uterus, are primarily regulated by TGF-β and IL-10 or by TGF-β and IL-15, respectively. Abbreviations: FL, fms-like tyrosine kinase 3 ligand; KL, kit ligand; IL, interleukin; HSC, hematological stem cell; CLP, common lymphoid progenitor; TGF-β, transforming growth factor-β.

Natural killer (NK) cells were first identified as “natural killer cells” in the mid-1970s and were characterized by their vital roles in controlling cancer and viral infection (1–3). They are widely distributed in diverse tissues, such as the peripheral blood (PB), spleen, lungs, liver, and uterus (4). In human PB, NK cells are primarily divided into two subtypes: CD3^−^CD56^dim^CD16^+^ and CD3^−^CD56^bright^CD16^−^ cells. CD56^dim^ NK cells have potent cytotoxicity and high CD16 expression, allowing them to induce antibody-dependent cell-mediated cytotoxicity (ADCC) toward target cells, whereas CD56^bright^ NK cells are best known for producing diverse types of cytokines (5–7). Different from PB NK cells, NK cells in diverse tissues have distinct phenotypes. Through experimental parabiosis (8), researchers have found that, with the exception of circulating NK cells, the identification of several markers, such as CD69, CD103, and CD49a, can affirm the phenotype of tissue-resident NK cells in the liver, skin, and uterus (4, 9–14). Functions of NK cells vary depending on the cellular microenvironment, mainly due to the cytokine signals of various tissues. For instance, NK cells can regulate the outcome of pregnancy (15, 16) through the regulation of transforming growth factor (TGF)-β and interleukin (IL)-15 in the uterus (17–19) or tolerate plentiful food-derived antigens or bacterial products through the regulation of abundant TGF-β and IL-10 in the liver (20–23) (Figure 1).

## Regulation of NK Cell Development by Cytokines

Natural killer cells can develop in many sites, including the fetal liver, bone marrow (BM), and thymus (24–27). The developmental hierarchy of NK cells has been depicted as a linear process that involves multiple stages, developing from hematopoietic stem cells (HSCs) through common lymphoid progenitors (CLPs) and NK lineage-restricted progenitors (NKPs) to mature NK cells (28). In contrast to the mouse, the human NK cell development hierarchy is less well characterized (29). Recently, human NKPs, which can exclusively differentiate into NK cells, have been clearly defined as Lin^−^CD34^+^CD38^+^CD123^−^CD45RA^+^CD7^+^CD10^+^CD127^−^ in umbilical cord blood (UCB), BM, and tonsils (30). The development of NK cells is regulated by cell-intrinsic signals through an array of transcription factors (TFs) and extrinsic signals from multiple cytokines (31–35). The TFs, including E4BP4, T-bet, Eomes, and GATA3 can regulate NK cell differentiation and maturation (26, 36–39). However, the requirement for TFs in murine NK cells may differ in different tissues. For instance, tissue-resident liver NK cells critically require the regulation of T-bet, whereas circulating NK cells are less affected by its depletion (12, 39, 40). Thymic NK cells depend on the regulation of GATA3, whereas circulating NK cells and tissue-resident liver NK cells do not (26). Nevertheless, further study of transcriptional regulation in the development of human NK cells is still required for a greater understanding of these processes. The extrinsic cytokine signals that are crucial for regulating NK cell development have been well characterized. Cytokines, such as fms-like tyrosine kinase 3 ligand (FL), kit ligand (KL), and IL-3, influence the survival, and proliferation of HSC, and are important for normal NK cell development (41, 42). In addition, NK cells are nearly absent in IL-15^−/−^ or IL-15Rα^−/−^ mice, which implies an indispensable role for IL-15 during NK cell differentiation (43, 44). Furthermore, previous reports have shown that IL-2^−/−^, IL-2Rα^−/−^, IL-7^−/−^, IL-7Rα^−/−^, and IL-21R^−/−^ mice have normal numbers of mature NK cells in PB, suggesting that IL-2, IL-7, and IL-21 are redundant for peripheral NK cell development (45–48). However, IL-2 and IL-21 participate in promoting NK cell activation with enhanced cytotoxicity (49–51). Additionally, IL-12, IL-18, IL-10, and TGF-β also have roles in NK cell development or function (33). In this review, we illustrate when and how each cytokine regulates NK cell development and function (Table 1). Based on a thorough understanding of relative cytokines, NK cells can be further generated in large quantities using diverse cytokine cocktails for *in vitro* expansion and induction in culture systems for adoptive transfer therapy.

**Table 1:** The receptors, signaling pathways, and knockout phenotypes of cytokines.

Cytokine	Receptors	Signaling pathways	Knockout phenotypes	Reference
SCF (KL)	KIT	PI3K-AKT JAK-STAT1/3/5 Ras-MEK-ERK	Deficiency of HSCs, mast cells, and NKPs	(52–56)

FLT3L (FL)	FLT3	PI3K-AKT JAK-STAT5 Ras-MEK-ERK	Deficiency of CLPs, DCs, and NK cells	(57–60)

IL-3	IL-3Rα/βc	PI3K-AKT JAK2-STAT1/3/5/6 Ras-MEK-ERK	Deficiency of mast cells, basophil cells, and embryonic HSCs	(61–63)

IL-7	IL-7Rα/γc	JAK1/3-STAT5 PI3K-AKT Normal NK cell number	Deficiency of B cells and T cells	(46, 64, 65)

IL-15	IL-15Rα/β/γc	JAK1/3-STAT5 PI3K-AKT-mTOR Ras-MEK-MAPK	Deficiency of NK, NKT, IEL, and memory CD8^+^ T cells	(43, 44, 66)

IL-2	IL-2Rα/β/γc	JAK1/3-STAT5 PI3K-AKT Ras-MEK-MAPK Normal NK cell number	Deficiency of Treg cells Accumulation of activated T/B cells	(45, 67–70)

IL-12	IL-12Rβ1/2	TYK/JAK2-STAT4 Failure of Th1 cell polarization	Reduced autoimmune diseases	(71–74)

IL-18	IL-18R1/Rap	MyD88-IRAK4-NF-κb	Impairment of Th1 cell polarization and NK cell cytotoxicity	(75–78)

IL-21	IL-21R/γc	JAK1/3-STAT3 PI3K-AKT Ras-MEK-MAPK	Normal NK cell number Decreased Th17 cells with reduced progression of EAE Impaired secretion of IgG	(48, 79–82)

IL-10	IL-10R1/2	TYK2/JAK1-STAT3/1/5	Activated CD4 T cells accumulation Dysfunction of Treg cells	(83–86)

TGF-β	TGF-βRI/II	Smad	Auto-reactivity of immune cells Die at early age	(87–90)

*SCF, stem cell factor; KL, kit ligand; FLT3L (FL), fms-like tyrosine kinase 3 ligand; IL, interleukin; TGF-β, transforming growth factor β; βc, common β chain; γc, common γ chain; Rap, receptor accessory protein; PI3K, phosphoinositide 3 kinase; AKT, protein kinase B; JAK, janus kinases; STAT, signal transduction and activation of transcription; ERK, extracellular regulated protein kinases; MAPK, mitogen-activated protein kinase; TYK, tyrosine kinase; MyD88, myeloid differentiation primary response protein 88; IRAK4, IL-1R-associated kinase 4; NF-κb, nuclear factor kappa-light-chain-enhancer of activated B cells; HSC, hematopoietic stem cell; DC, dendritic cell; NK, natural killer; NKT, natural killer T; IEL, intraepithelial lymphocytes; Treg, regulatory T cell; Th, T helper; EAE, experimental autoimmune encephalomyelitis*.

Natural killer cells belong to the innate immune system due to their roles in directly combating hematopoietic and non-hematopoietic cells to maintain homeostasis throughout the body (91). Previous reports have divided the developmental pattern of NK cells into four stages based on expression of the cell surface markers CD34, CD117 and CD94:CD34^+^CD117^−^CD94^−^, CD34^+^CD117^+^CD94^−^, CD34^−^CD117^+^CD94^−^, and CD34^−^CD117^+/−^CD94^+^ (92). NK cell development and function are characterized by the gradual acquisition of specific receptors. As shown above, the extrinsic signals from cytokines are vital important to regulate NK cell development and function (31–33). According to the expression pattern of cytokine receptors, we have divided NK cell development into five grading stages, including HSCs: CD34^+^CD117^+^FLT3^+^CD123^+/−^, CLPs: CD34^+^CD117^+^FLT3^+^CD123^−^CD127^+^, NKPs: CD34^+^CD117^+^FLT3^+^CD7^+^CD10^+^CD122^+^CD127^−^CD215^+^, immature NK cells (iNK cells): CD56^+/−^CD25^+^CD122^+^CD212^+/−^CD215^+^CD218^+/−^CD360^+/−^, and mature NK cells (mNK cells): CD56^+^CD25^+/−^CD122^+^CD212^+^CD215^+^CD218^+^CD360^+^TGF-βR^+^ IL-10R^+^ (Figure 1). The differential expression of cytokine receptors implies that there are various demands for the relevant cytokines during NK cell development.

### FL, KL, IL-3, and IL-7 Promote the Transition of HSCs to CLPs

Hematological stem cells are the major source of multilineage myeloid cells and lymphocytes which are vital for maintaining normal numbers and functions of immune cells (93). It is well established that the differentiation of NK cells is a step-wise process that is driven by the regulation of TFs and coordinated cytokine signals from HSCs (31–35). FL and KL, discovered in the early 1990s, have overlapping yet distinct effects to promote HSC survival and proliferation (41). Their receptors, flt3 and c-kit, belong to the family of tyrosine kinase receptors expressed primarily on cells in the very early stages of hematopoiesis (54, 57, 94). KL, also known as stem cell factor (SCF), is produced in two forms: membrane-bound and soluble, through differential splicing and proteolytic cleavage (53, 95). *Sl*/*Sl*^d^ mutant mice expressing only soluble SCF are deficient in HSCs, indicating that membrane-bound SCF is critical for HSC maintenance (52, 55, 96, 97).

Previous reports have shown that flt3 or c-kit deficiency in mice induces a reduction in the number of CLPs (58, 98). In addition, the cytokine FL deficiency in mice can also induce a sharp reduction of CLPs but has no effect on the HSC pool or on common myeloid progenitors (59, 60). Recently, it is revealed that FL can act synergistically with Hoxa9 signaling to regulate an early checkpoint of lymphopoiesis by affecting CLPs, LMPPs, and flt3^+^ multipotent hematopoietic progenitors in the BM (99). Furthermore, FL and SCF can act synergistically to promote CD34^+^ cell proliferation and are important for NK cell differentiation by inducing the expression of CD122 and increasing *IL15R*α expression to increase their sensitivity to IL-15 (100). The number of NKPs is reduced in mice that are deficient in either flt3 or c-kit, implying that either of these cytokines is essential for NK-cell differentiation (56). These findings imply that FL and SCF have important roles in the development of lymphoid progenitor cells and are critical for the commitment of NK-cell lineage.

Interleukin-3 is a member of the beta common (βc) family of cytokines, which also includes granulocyte-macrophage colony-stimulating factor (GM-CSF) and IL-5 (101). IL-3 is a well-known hematologic growth factor that can promote HSC survival, proliferation and differentiation (62). Previous research has demonstrated that IL-3 alone can rescue low HSC activity in *Runx1^+/−^* AGMs (Aorta-Gonad-Mesonephros) that have a reduced HSC population (63, 102). Receptors for IL-3, members of the gp140 family, are composed of an IL-3 receptor-specific α subunit (IL-3Rα or CD123) and a homo-dimeric βc subunit (61, 103). Both CD123 and βc subunits are detected on the surface of hematopoietic tissues and HSCs (42). After binding with the receptors, it can activate janus kinases (JAK) 2-signal transduction and activation of transcription (STAT) 5/1/3/6, phosphoinositide 3 kinase (PI3K)-protein kinase B (AKT), and Ras-extracellular regulated protein kinases (ERK) pathways (62, 104). In the *in vitro* differentiation system of human primitive progenitors, IL-3 has been reported to maintain lymphoid progenitor development and promote NK cell or B cell differentiation (105–107). Moreover, IL-3 can also preserve the engraftment and lymphoid reconstitution capacity *in vivo* of the transduced CD34^+^ cells in severe combined immunodeficiency (SCID)-hu mice (108). Therefore, IL-3 may primarily facilitate the survival and proliferation of HSCs and the differentiation of CLPs, and further promote NK cell development.

CXCR4 signaling has been shown to regulate quiescence and long-term maintenance of HSCs upon interaction with the chemokine CXCL12 (109, 110). Recently, a group of researchers found that CXCR4 can provide lineage-instructive signals to control progenitor cell differentiation (111). They showed that signals from CXCR4-CXCL12 interactions regulate multipotent progenitor (MPP) differentiation into CLP subsets in the BM and further affect lymphoid lineage production. Moreover, a deficiency of CXCR4 signaling resulted in a profound reduction in the number of T, B, and NK cells which suggests that the addition of CXCL12 may be helpful to promote *in vitro* NK cell differentiation from HSCs.

Interleukin-7 is another important cytokine for the differentiation of lymphoid lineages, mainly for the differentiation of T and B cells (46, 64). It induces the differentiation of HSCs into lymphoid progenitor cells and facilitates their expansion and survival. The IL-7 receptor is a heterodimeric complex composed of IL-7Rα (CD127) and the common γ chain subunit (CD132) (112). The IL-7-IL-7R interaction primarily activates JAK1/3-STAT5 and PI3K-AKT pathways to induce prosurvival, cell cycle, and metabolism regulation signals (65, 113). Previous reports have shown that knockouts of IL-7 and IL-7Rα do not induce significant defects in mouse NK cells from the PB or spleen (46, 47). Thus, IL-7 may contribute in a redundant way and may not be essential for circulatory NK cell development. However, NK cells in the thymus, characterized by IL-7Rα^+^, require IL-7 for their homeostasis (26). Whether other NK cell subsets in different tissues require IL-7 for their effector functions or homeostasis is unknown.

### IL-15 Directs CLPs toward Mature NK Cells

Important cytokines for the development and function of immune cells are highlighted in X-SCID, characterized by mutations of *IL-2RG*, which encodes the common γ chain (γc), a common receptor for IL-2, IL-4, IL-7, IL-9, IL-15, and IL-21 (114, 115). X-SCID is characterized by extreme vulnerability to viruses and pathogens due to the developmental and functional deficiency of T, B, and NK cells, which indicates that γc family cytokines play vital roles in normal immune responses.

IL-15^−/−^ and IL-15R^−/−^ mice have dramatically reduced populations of NK cells (43, 44); however, IL-2^−/−^Rag1^−/−^, IL-7^−/−^, and IL-21R^−/−^ mice have normal NK cell numbers (45–48), suggesting that IL-15 is essential for NK cell commitment and maturation. Furthermore, mature NK cells fail to be maintained when transferred to IL-15^−/−^ mice (116, 117). The prosurvival ability of IL-15 is potentially mediated by upregulated expression of antiapoptotic B cell lymphoma 2 (BCL-2) family members and downregulated expression of proapoptotic proteins (116). IL-15 has extensive roles in immune cells. For example, it can regulate NKT cell development and maintain normal memory phenotypes of CD8^+^ cells (43, 44, 117–120).

Interleukin-15 can be produced by hematopoietic and non-hematopoietic cells, such as activated DCs, macrophages, monocytes, and stromal cells (121). IL-15R is a heterotrimeric receptor including a unique IL-15Rα (CD215) subunit, a shared β chain with IL-2 (CD122), and CD132 (122, 123). The expression of CD122 is a major phenotypic marker of NKPs, which allows the cells to respond to IL-15 (124, 125). IL-15 has a special manner of transducing its signals through trans-presentation, whereby the IL-15-producing cells support IL-15 by binding IL-15Rα, and then present it to activate neighboring cells expressing CD122/CD132 (126–130). Enlightened by these findings, researchers have designed NK cells expressing membrane-bound IL-15 that have autonomous growth and increased cytotoxicity toward tumor cells, which can help to enhance the antitumor effects of NK cells and avoid the side effects of cytokine administration (131). The activation of IL-15R induces the autophosphorylation and activation of JAK1/3 and downstream cascades, including the JAK-STAT, the mitogen-activated protein kinase (MAPK), and PI3K/AKT-mTOR signaling pathways, in order to fulfill its different functions (66, 132). The IL-15-STAT5 signaling pathway is indispensable for NK cell development and homeostasis, as mice that are deficient in this pathway have dramatically reduced mature NK cell numbers. In addition, both *Stat5*-deficient and NK cell-specific *Stat5*-deficient mice have an absence of NK cells in peripheral blood and tissues (133–135). A patient with a *STAT5b* mutation also showed a severe reduction in NK cell numbers (136). The PI3K/AKT-mTOR pathway also plays a role in NK cell development. A recently published paper has shown that PDK1, a kinase upstream of mTOR, is a critical component that connects IL-15 signaling to E4BP4, an indispensable TF for NK cell development (137). The early depletion of PDK1 induces a severe loss of NK cells with much weaker mTOR activation, E4BP4 induction after IL-15 stimulation and the reduced expression of CD122 (137). These findings underscore the importance of the IL-15-PI3K-PDK1-mTOR-E4BP4-CD122 positive feedback loop in the development of NK cells. Other factors can also affect NK cell development by influencing their responsiveness to IL-15. The TF ID2 can affect NK cell development by antagonizing E-protein function and altering lineage fate (138, 139). Recently, researchers have found that ID2 can suppress E-protein target gene SOCS3 expression to maintain IL-15 receptor signaling for normal NK cells development, and strong IL-15 receptor stimulation can overcome this requirement for ID2 (140). The abovementioned findings strengthen the roles of IL-15 in NK cell development and explain how IL-15 induces its effects.

## Polarization of NK Cell Function by Cytokines

Natural killer cells have diverse functions in different tissues, which can be divided into three subsets: cytotoxic, regulatory, and tolerant NK cells (141). Different NK cell subsets are primarily affected by signals from diverse cellular microenvironments and signals from cytokines are important in shaping their unique functions. In an inflammatory or virus-infected situation, activated T cells and dendritic cells (DCs) secrete abundant IL-2, IL-12, IL-18, and IL-21, which drive NK cell activation with enhanced secretion of cytotoxic cytokines and the ability to directly kill transformed targets (142–144). As mentioned above, NK cells in their physical environment play leading roles in maintaining normal pregnancy in the uterus and tolerating a significant amount of food-derived or gut-derived antigens in the liver, which are mainly determined by the regulation of different cytokine cocktails.

### IL-2 and IL-15 Induce NK Cell “Priming”

Natural killer cells serve as the first line of defense during viral infection or the elimination of transformed cells, which is characterized by the production of cytokines, such as IFN-γ and TNF-α, and granzyme-mediated cytotoxicity (3). Comprehensive activation is necessary for NK cells to fulfill their roles as sentinels (145). Previous reports have demonstrated that IL-2, IL-15, IL-12, IL-18, and IL-21 all play roles in activating NK cells. Therefore, we discuss how these cytokines function and how to achieve better efficacy in adoptive NK cell therapy.

Interleukin-2 is an immune-stimulatory cytokine that was first identified as a “T cell growth factor” (67–69, 146). Subsequently, it has been shown that activated T cell-derived IL-2 can enhance NK cell responses toward infection *in vivo* and can activate NK cells *in vitro* (142, 147). Furthermore, researchers found that NK cell responses are impaired in IL-2β^−/−^ mice suggesting that IL-2 may have an important role in maintaining NK cell activity (148). However, IL-2-deficient mice have normal NK cell development and numbers, which implies that it is needed for NK cell effector functions but is not indispensable for their development (45). IL-2 achieves its functions predominantly through the JAK1/3-STAT5 signaling pathway by binding with the heterotrimeric receptor, which is composed of IL-2Rα (CD25), CD122, and CD132 (70). Interestingly, IL-2 has different affinities for the different receptors. IL-2 binding to a single CD25 (Kd = 10^−8^ M) or CD122 (Kd = 10^−6^ M) has a low affinity. There is an intermediate binding affinity with the CD25/CD122 heterodimer (Kd = 10^−9^ M), and the greatest binding affinity is seen with the IL-2Rαβγ trimeric complex (Kd = 10^−11^ M) (149–151). NK cells primarily express CD122 and CD132 receptor components that can respond to high concentrations of IL-2. To achieve better IL-2 application efficacy, one group of researchers developed an IL-2 “superkine,” also called super-2, with increased affinity for CD122 (152). Super-2 has a vigorous role in promoting activation and proliferation irrespective of CD25 expression. In addition, previous reports have shown that preactivation with IL-12, IL-15, and IL-18 induces CD25 expression on NK cells, which suggests that pretreatment of NK cells with the above cytokines can enhance NK cell responsiveness to IL-2 and may lead to better IL-2 treatment efficacy (153, 154). However, high-dose IL-2 induces a selective expansion of regulatory T (Treg) cells, which limits the activity of NK cells, resulting in poor clinical responses to IL-2 therapy (155). The depletion of Treg cells leads to increased IL-2 availability for NK cells to increase IFN-γ production and cytotoxicity (155, 156). To overcome Treg cell inhibition, researchers have produced a mutant form of IL-2 that preferentially binds to CD122/CD132 and that has reduced binding to CD25 (157). In contrast to wild-type IL-2, the mutant form efficiently induces NK cell proliferation and activation with a dramatic reduction in Treg cells, and achieves better responses to tumors.

Interleukin-15 is discovered by its “IL-2-like” stimulatory role and is important for NK cell development and function (158–160). IL-15 and IL-2 share the common β and γ chain receptor subunits and only differ due to an α chain, as IL-2Rα (CD25) binds to IL-2 and IL-15Rα binds to IL-15 (122, 123, 161, 162). However, IL-15Rα alone binds to IL-15 with a high affinity (Kd = 10^−11^ M), which is comparable to that of the binding of IL-2Rαβγ to IL-2 (162). The high affinity between IL-15 and IL-15Rα makes it possible, when compared with IL-2, to activate NK cells with relatively lower concentrations.

Prior exposure to IL-15 sensitizes NK cells to secondary stimuli, referred to as “priming,” thereby resulting in exaggerated responses (163, 164). Previous studies have shown that IL-12 can induce elevated IFN-γ production in IL-15-primed NK cells. Other cytokines, such as IL-2, IL-4, IL-21, and type I IFN, can also induce heightened functions (165). As mentioned above, the JAK-STAT5 signaling pathway is important for IL-15-mediated NK cell development and viability (133–136). However, the PI3K-AKT-mTOR signaling pathway is critical for the IL-15-induced priming effect (132, 165). Inhibition of the PI3K-mTOR signal would abrogate enhanced IL-12- and IL-21-induced phosphorylation of STATs and stronger responses in IL-15-primed NK cells (165). Nevertheless, the inhibited mTOR pathway does not affect the phosphorylation of STATs in naïve NK cells, suggesting that this pathway specifically functions in IL-15-primed NK cells (165). The crosstalk between the PI3K-mTOR signaling pathway and the STAT pathway is critical for efficient NK cell priming; however, the mechanism by which this occurs remains unclear.

A previous report has shown that IL-15 promotes NK cells to be fully equipped to respond to infections through the rapid induction of granzymes and perforin (166). However, the induced production of granzymes/perforin is reduced in NK-mTOR^−/−^ cells, suggesting that mTOR is significant for mediating NK cell cytotoxicity (167). Researchers have also shown that IL-15, but not IL-2, can maintain NK cell cytolytic functions after cytokine withdrawal to simulate postinfusion performance (168). The IL-15-induced functional advantages are dependent on activated mTOR-regulated signaling (168). The mTOR-regulated IL-15-induced maintenance of NK cell functions suggests a beneficial implementation of IL-15 in adoptive NK-cell clinical therapy.

### IL-12, IL-18, and IL-21 Strengthen NK Cell Cytotoxicity

Interleukin-12 was first named NK cell stimulating factor based on its ability to induce NK cells to secrete high levels of IFN-γ (169). It is composed of two subunits, IL-12p35 and IL-12p40, and these two subunits must be coexpressed in the same cell to form the disulfide-linked bioactive IL-12p70 (170). The cytokine is mainly produced by antigen-presenting cells, such as DCs, monocytes and macrophages (171–173). NK cells and activated T cells express the high-affinity heterodimeric receptor (IL-12Rβ1/β2) for interaction with IL-12, which can activate tyrosine kinase 2 (TYK2) and JAK2, leading to phosphorylation of STATs (mainly STAT4) and the eventual promotion of IFN-γ production and other biological reactions (71–74, 174).

One previous report suggested that NK cells can mediate a long-lived contact hypersensitivity response to haptens in mice devoid of T and B cells; thus, the concept of “memory NK cells” was established (175). However, the mechanism by which memory NK cells develop is unknown. The group of Lewis L. Lanier observed that IL-12-STAT4-dependent-IFN-γ-independent signaling is indispensable for the generation of mouse cytomegalovirus-specific memory NK cells (176). As memory NK cells are long-lived and have relatively higher responses compared with naïve NK cells (175, 177–179), developing memory NK cells for clinical therapy is an attractive approach. Wayne M. Yokoyama’s group first showed that preactivation of mice NK cells with IL-12 and IL-18, along with low-dose IL-15 to maintain survival, could produce cytokine-induced memory-like NK cells (180). The memory-like cells could respond more robustly to reactivation without inducing enhanced cytotoxicity toward tumor cells (180). However, Adelheid Cerwenka laboratory observed that IL-12/15/18 preactivated NK cells, when combined with irradiation, could achieve greater efficacy, as determined by reduced growth of established mouse tumors (153). They also showed that the antitumor effect is mediated by IL-2 produced by CD4^+^ T cells. As the abovementioned memory-like NK cells were all established in mice, Romee et al. showed that human NK cells can also display memory characteristics after short-term preactivation with IL-12/15/18 (181). Subsequently, they also found that the memory-like NK cells induce prolonged expression of CD25, forming a high affinity IL-2R complex to respond to IL-2 at picomolar concentrations (154). However, single cytokine, like IL-12, IL-15, or IL-18 preactivation cannot induce a higher expression of CD25. Moreover, prior treatment with low-dose IL-2 before adoptive transfer can enhance proliferation and effector function of memory-like NK cells, which supports an additional immunotherapy strategy. Recently, this group performed a phase 1 study to treat active rel/ref AML patients with memory-like NK cells (182). They found that donor memory-like NK cells exhibited enhanced IFN-γ production and yielded an overall response rate of 55%. This result reminds us that preactivation with cytokines can indeed strengthen NK cell antitumor functionality and can be used as a therapeutic method to treat tumors. Nevertheless, understanding the appropriate cell doses still requires further study.

The NK cells without expressing any inhibitory MHC-I-specific receptors, such as killer cell immunoglobulin-like receptors (KIRs) in humans and Ly49 receptors in mice, are hyporesponsive. The signals from KIRs and Ly49 receptors are critical for NK cells to be functionally competent (183, 184). This process is also termed “NK cell licensing.” A recent report has shown that the unlicensed NK cells display enhanced functionality after preactivation with IL-12, IL-15, and IL-18 (185). It has also been reported that human CD56^bright^KIR^−^ and CD56^dim^KIR^−^ NK cells can acquire KIR expression upon stimulation with IL-15 in the presence of stromal cells (186). Furthermore, the developed KIR^+^ NK cells display enhanced cytotoxicity and cytokine-producing potential compared to the KIR^−^ NK cells. Later, another group has identified that activation with cytokines such as IL-2, IL-15, or IL-12 can induce the *de novo* expression of KIR and/or NKG2A on KIR^−^NKG2A^−^ NK cells without feeder cells (187, 188). Similar to human NK cells, the responsive capacity of unlicensed murine NK cells can be restored as licensed cells when stimulated *in vitro* with high doses of IL-12 and IL-18 or IL-2 (183). These findings suggest that cytokine stimulation can induce the hypo-responsive unlicensed NK cells to be re-educated and acquire stronger responses toward target cells.

Interleukin-12 has also been shown to promote further maturation of *in vitro-*differentiated NK cells with enhanced cytotoxicity. One group has shown that low-dose IL-12 can decrease the fluorescence intensity of CD56 and can induce the expansion of more mature CD56^dim^CD16^+^ and CD56^dim^KIR^+^ NK cells (189). Subsequently, another group identified that *in vitro*-derived NK cells display enhanced cytotoxicity toward primary AML cells and have improved antileukemic responses in MHC class I-positive AML mice after IL-12 culturing (190). These findings suggest that IL-12 can not only regulate the functions of mature NK cells but also promote the differentiation level and function of developing NK cells.

Interleukin-18 is a proinflammatory cytokine belonging to the IL-1 cytokine family and was originally defined as an IFN-γ-inducing-factor (191, 192). It is constitutively produced by hematopoietic cells, such as DCs, macrophages and neutrophils (191, 193–195), and non-hematopoietic cells, such as microglial cells and epithelial cells (196). IL-18 is initially produced as an inactive precursor, pro-IL-18, which requires cleavage by caspase-1 of the N-terminal fragment to become the mature, biologically active form (197, 198). Mature IL-18 binds to its receptor, composed of IL-18R1 and IL-18R accessory protein in a heterodimeric receptor complex, to initiate signal transduction by myeloid differentiation primary response protein 88 (MyD88). Then, IL-1R-associated kinase 4 (IRAK4) and TNFR-associated factor 6 (TRAF6) are recruited, leading to the activation of the nuclear factor (NF) kappa-light-chain-enhancer of activated B cells (κB) and MAPK pathways to promote IFN-γ transcription and stabilization of *IFNG* mRNA (78, 199).

Interleukin-18 is important for promoting the production of IFN-γ by NK cells against viral, fungal, bacterial, and parasitic infections (200–203). NK cells display reduced secretion of IFN-γ and compromised cytotoxicity in IL-18-deficient, IL-18R1-deficient, or IRAK4-deficient mice (75–77). Furthermore, IL-18 is essential for upregulating CD25 expression in NK cells, and the increased production of IFN-γ is enhanced by IL-2 during *Plasmodium yoelii* infection in mice (204). Similarly, low-dose IL-18, through synergistic interactions with IL-2, IL-12, IL-15, or IL-21, can induce potent CD25 and IFN-γ upregulation to strengthen human NK cell effector function (205). A previous report has shown that T and B cells can upregulate IL-18R expression upon IL-12 treatment, and the combination of IL-18 and IL-12 synergistically induces their IFN-γ production (206). Recently, researchers also found that the combination of these two cytokines can reverse NK cell anergy and increase the survival rate of mice bearing MHC-deficient tumors (207). These findings imply that IL-18 can play stronger roles when combined with other stimulatory cytokines; therefore, combining cytokines together is better for achieving enhanced efficacy.

Interleukin-21 is a pleiotropic cytokine mainly produced by T follicular helper cells, Th17 cells, and NKT cells (208). It acts through a receptor complex, including IL-21R and γc (209, 210), to activate JAK1 and JAK3, which leads to recruitment and phosphorylation of STAT (predominantly STAT3 but also STAT1 and STAT5) to promote the expression of IFN-γ and other factors (81). The IL-21 signal can also be transduced by the MAPK and PI3K/AKT pathways (81).

Interleukin-21 has diverse effects in immune cells, which can affect the differentiation of inflammatory T cells, immunoglobulin production of B cells, and development and functions of NK cells (48, 79, 80, 82, 211). IL-21, combined with FL and IL-15, can specifically promote the differentiation and expansion of CD16^+^CD56^+^ cytotoxic NK cells from BM progenitors *in vitro* (210). It also promotes rapid differentiation and acquisition of killer Ig-like receptors of NK cells from cord blood CD34-positive cells (212). Additionally, it induces mature mouse NK cells to develop a large granular lymphocyte phenotype with increased production of cytokines, such as IFN-γ, and perforin through coactivation with IL-2 or IL-15, resulting in enhanced cytotoxicity (213). Similarly, human NK cells cocultured with IL-21 and therapeutic antibody-coated breast cancer cells secrete higher levels of IFN-γ, TNF-α, IL-8, CCL3, and CCL5, and the supernatants are able to drive the migration of naïve and activated T cells *in vitro* (214). The administration of IL-21 and antibody-coated tumor cells leads to synergistic cytotoxic effects of NK cells toward tumor cells, which suggests that IL-21 may be an effective adjuvant for antibody treatment (214). Interestingly, IL-21 limits IL-15-mediated NK cell expansion and viability; nevertheless, it can stimulate IFN-γ production and cytotoxicity in NK cells previously activated with poly I:C or IL-15 (48). This finding reminds us that it may be important to apply cytokine cocktails sequentially.

Insulin-like growth factor 1 (IGF-1) is an important growth factor to regulate longevity and immunity (215). And it was also shown to promote NK cell development from CD34^+^ cells and increase NK cell cytotoxicity by promoting the production of perforin by STAT3 activation (216). Furthermore, foxO1, which negatively regulates NK cell maturation and function, is a key molecule of IGF-1 signaling pathway (217, 218). Therefore, IGF-1 may also strengthen NK cell function through IGF-1-induced foxO1 inactivation, which is mediated by increased phosphorylation of foxO1 at Ser256 and Thr24 (219). Such information about IGF-1 may present new opportunities to boost NK cell cytotoxicity therapeutically.

### TGF-β and IL-10 Shape Tolerant NK Cells

Natural killer cells are not homogenous, and cells with low cytotoxicity, residing in the liver or a chronic pathogenic microenvironment, are termed tolerant NK cells (141). The liver is an important immune-tolerant organ, as no severe inflammation occurs despite constant stimulation by bacterial products and antigens from the gut (20, 220, 221). NK cells, composed of CD56^dim^ and CD56^bright^ subtypes, occupy up to 30–40% of all hepatic lymphocytes located in human liver sinusoids (222, 223). CD56^dim^ hepatic NK cells share many similarities with PB CD56^dim^ NK cells that may frequently circulate throughout the body. By contrast, CD56^bright^ cells with CD69 and CD49a expression are more specifically retained in the liver (13, 224). Similarly, one subset of DX5^−^CD49a^+^ mouse NK cells specifically resides in the liver with adaptive-like properties (10). However, the specific roles of CD56^bright^ and CD56^dim^ human intrahepatic NK cells still require further study. One important function of intrahepatic NK cells is to tolerate harmless antigens that can help maintain liver homeostasis (225). IL-10 and TGF-β, which are produced by DCs, Kupffer cells, hepatic sinusoidal endothelial cells, and stellate cells, are important for shaping tolerant NK cells.

Interleukin-10, first recognized for its ability to inhibit the activation and cytokine secretion of Th1 cells, is described as a cytokine synthesis inhibitory factor (83–85, 226). It is an important immune-regulatory cytokine that is produced by T cells, B cells, NK cells, DCs and macrophages (227, 228). The receptors for IL-10 mainly contain two subunits, IL-10R1 and IL-10R2, and are expressed on many hematopoietic and non-hematopoietic cells (229). Once IL-10 and IL-10R bind together, JAK1 and TYK2 are activated, which leads to the phosphorylation and activation of STAT3, STAT1, and STAT5 (86). As mentioned above, IL-10 is important for allowing liver NK cells to maintain immune-tolerant states. Indeed, one study demonstrated that intrahepatic IL-10 can maintain NK cells in a functionally hypo-responsive state with a phenotype of NKG2A^+^Ly49^−^ cells (22). NKG2A is critical for NK cells, when cocultured with non-transformed hepatocytes, to prime DC cells. Furthermore, the primed-DCs can result in the induction of regulatory CD4^+^CD25^+^ T cells, a subset responsible for inhibiting excessive immune activation (230, 231). Even transferred splenic NK cells migrating into the liver show changes in phenotype and function, suggesting that the liver environment reshapes NK cells to maintain a steady state (22).

Transforming growth factor-β includes three isoforms, TGF-β1, 2, and 3, which are highly homologous (232). TGF-β1 is the primary isoform expressed in the immune system (233). If deficient, mice die of systemic inflammation by 3–4 weeks of age, suggesting that TGF-β plays important roles in maintaining normal immune responses (87–89). TGF-β binds to its receptors, primarily TGF-βRI and TGF-βRII, to activate a downstream cascade (234). First, the intracellular receptor Smad (R-Smad) proteins Smad2/3 are recruited and phosphorylated, and the phosphorylated Smad2/3 then combine with Smad4 or TIF1γ to form a trimeric complex. The trimeric complex translocates to the nucleus to regulate relative gene expression by binding to the responsive regulatory regions (90). TGF-β can also activate Smad-independent pathways, such as small GTPases, MAPK, and PI3K pathways (235).

Tolerant NK cells can mediate benign effects to maintain physiological homeostasis. However, cells induced in the context of chronic infection or the cancer microenvironment are harmful for the treatment of related diseases (236, 237). For example, TGF-β upregulation induces reduced secretion of IFN-γ by repressing the normal expression of T-bet in the pathologic niche (238). High levels of TGF-β induce a weak NKG2D/DAP10 and CD244/SAP signal, leading to NK cells that are unable to eliminate hepatitis B virus (HBV) infection in chronic hepatitis B (CHB) (239, 240). Later, another report showed that TGF-β and IL-10 in CHB-infected and hepatic carcinoma patients induce the expression of microRNA (miR)-146a, which causes reduced IFN-γ production and cytotoxicity, resulting in a poorer prognosis (241). Furthermore, TGF-β-induced miR-183 represses DAP12 transcription and translation (242), or reduces the expression of NKp30 and NKG2D (243), to further weaken NK cell cytotoxic functions in the tumor microenvironment. The abnormal tolerant NK cells cannot eliminate infected or transformed cells, which leads to immune evasion by these cells.

### TGF-β and IL-15 Develop Regulatory NK Cells

Decidual NK cells (dNK) are distinguishable from PB NK cells because more than 90% of these cells are characterized by a CD56^bright^CD16^−^CD49a^+^CD9^+^ phenotype with a low cytotoxic effect and high cytokine secretion ability (15, 244, 245). Previous reports have shown that they constitute 50–90% of decidual lymphocytes during the first trimester of pregnancy (244, 246, 247). The accumulated NK cells can control extravillous trophoblast invasion and vascular remodeling by secreting different amounts of molecules, such as GM-CSF, colony-stimulating factor 1, angiopoietin-2, vascular endothelial cell growth factor, and placental growth factor (248, 249). Furthermore, dNK cell-derived IFN-γ has been shown to be critical for vessel modification and decidual cellularity in mice (250). Inflammatory responses in decidual, caused by allogenic fetal cell invasion and other factors, can result in abortion (246, 251). To ensure a normal pregnancy, researchers have determined that regulatory T cells can mediate an inhibitory response to the aggressive alloantigen (252), and CD86 blockage (253) and PD-1 (254) involvement can both play protective roles in pregnancy outcomes. As the dominant member of decidual lymphocytes, dNK cells can provide immune-regulatory mechanisms to maintain a regular uterine environment. Indeed, IFN-γ derived from CD27^+^CD56^bright^ dNK cells dampens Th17-induced inflammation to maintain a normal pregnancy (255). The abnormalities in the proportion and IFN-γ secretion of dNK cells in patients with recurrent spontaneous abortions result in long-term Th17-induced inflammation and eventual pregnancy failure (255). Another study subsequently showed that the crosstalk between dNK cells and CD14^+^ myelomonocytic cells results in the generation of regulatory T cells for the inhibition of abnormal inflammation in the uterus (256).

The above findings demonstrate the important regulatory roles of dNK cells; however, whether dNK cells are derived from the differentiation of local NKPs or the migration of PB NK cells or both still needs to be studied. Nevertheless, under hypoxic conditions, endometrium-derived TGF-β and stromal cell-derived IL-15 may function to shape unique dNK cells (16, 19, 257). Indeed, TGF-β promotes the conversion of CD16^+^ PB NK cells to CD16^−^ cells and inhibits the expression of NKp30 to further inhibit cytotoxic functions, both of which lead to cells with similarities to dNK cells (17). Furthermore, TGF-β can induce the expression of CD103 and CD49a to increase the likelihood of the cells residing in the uterus (9, 258). Moreover, one study applied TGF-β1, IL-15 and a demethylating agent under hypoxic conditions to successfully transform PB NK cells into dNK-like cells (18). These findings imply that the specific hypoxic state of the uterus, combined with the help of TGF-β and IL-15, shapes particular regulatory NK cells.

## Cytokine Cocktails Promote *In Vitro* Expansion of NK Cells

**Figure 2:**
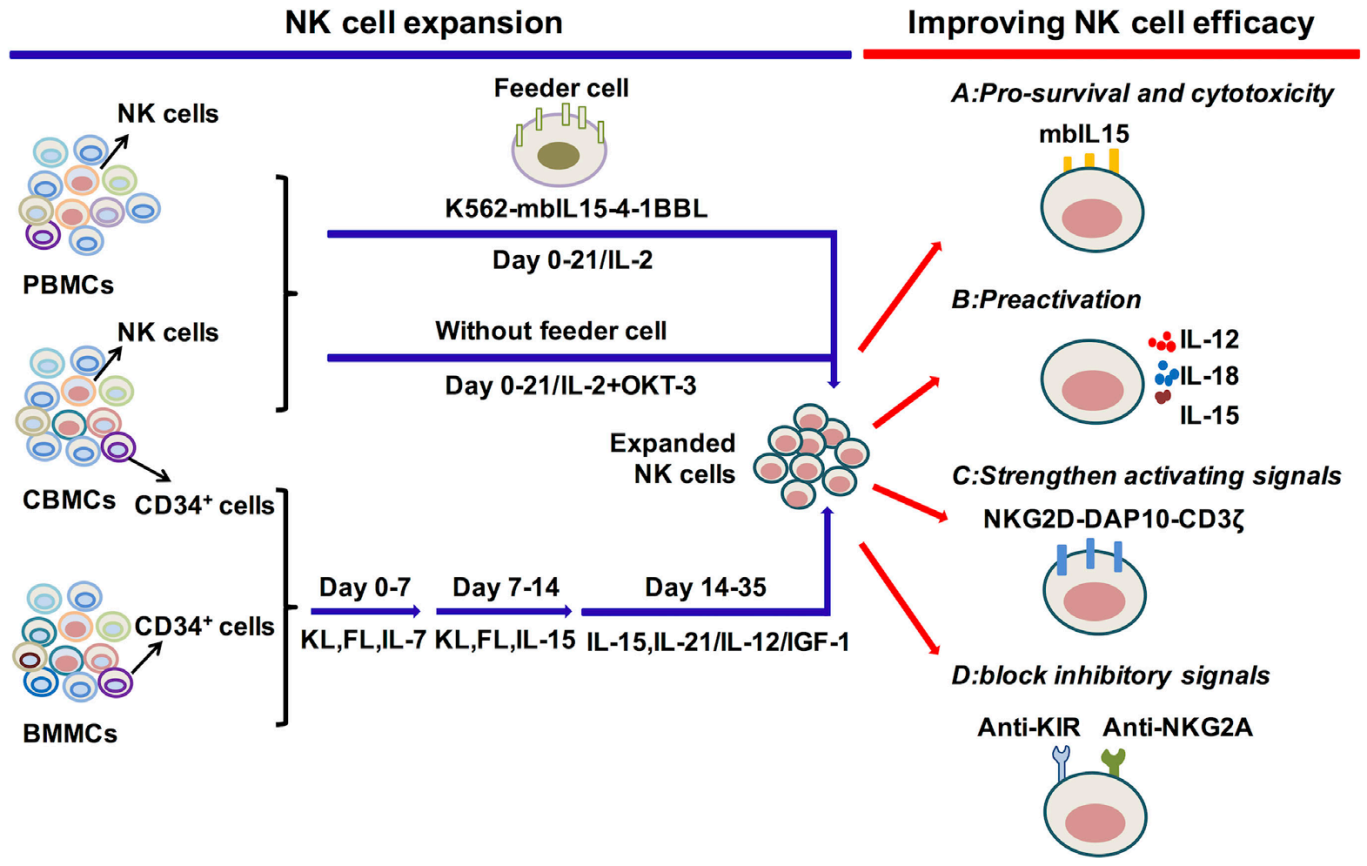
Cytokine regulation of natural killer (NK) cell expansion and cytotoxicity. Genetically modified K562 cells and IL-2 or IL-2 and OKT-3 without feeder cells, applied for the expansion of primary NK cells, can generate significant amounts of functional NK cells. The differentiation and expansion of NK cells from CD34^+^ HSCs are regulated by early activating cytokines, such as FL, KL, and IL-7, to promote HSC proliferation and differentiation, as well as by cytokines to activate NK cells, such as IL-15, IL-12, IL-21, and IGF-1. To improve NK cell survival or antitumor function, relative signals, such as the expression of mbIL-15 or preactivation with IL-12/15/18, strengthen activating or block inhibitory signals. These are vital for improving NK cell efficacy in adoptive cell therapy. Abbreviations: PBMC, peripheral blood mononuclear cell; CBMC, cord blood mononuclear cell; BMMC, bone marrow mononuclear cell; IL, interleukin; mbIL-15, membrane-bound IL-15; 4-1BBL, 4-1BB ligand; IGF-1, insulin-like growth factor 1; NKG2D, natural killer group 2D; DAP10, DNAX-activating protein 10; KIR, killer cell immunoglobulin-like receptors; NKG2A, natural killer group 2A.

As mentioned above, multiple cytokines can regulate NK cell development, proliferation and activation. Previous studies have observed that NK cells can exert graft-versus-leukemia reactions without causing graft-versus-host disease (GVHD) in allogeneic hematopoietic transplantation (259). Furthermore, pioneering work by the Miller group showed that infusion of haplo-identical NK cell infusions activated by IL-2 can induce remission in AML patients (260). NK cells, therefore, are promising candidates for the treatment of hematological malignancies. However, the generation of sufficient NK cell quantities with robust effectiveness remains challenging. Therefore, strategies to expand or induce NK cells from primary NK cells or CD34^+^ cells with high cytotoxicity using different combinations of cytokines are actively being developed (261, 262) (Figure 2).

### Expansion of NK Cells from PB or UCB NK Cells

The most effective protocols to expand NK cells from primary NK cells depend on the presence of feeder cells, such as genetically modified K562 cells, Epstein-Barr virus-transformed lymphoblastoid B cell lines (EBV-BLCL) and irradiated autologous cells (263–265) (Figure 2). One group has utilized double-transduced K562 cells with membrane-bound IL-15 and costimulating ligand 4-1BBL (K562-mbIL15-41BBL) as feeder cells along with low concentrations of IL-2, which results in dramatic PB NK cell expansion with negligible T cell expansion (264, 266, 267). K562-mbIL15-41BBL cell-stimulated NK cells can continually proliferate for 8–12 weeks, acquire up to 10^8^ percent of the number of originally seeded NK cells and maintain high cytotoxic capacity (268). The K562-mbIL15-4-1BBL-based expansion method has been adapted for use under Good Manufacturing Practice conditions that can be used in the clinic (264, 269). K562-mbIL-15-4-1BBL cells can also be used to expand UCB NK cells with enhanced proliferation and cytotoxicity (270). Other gene-modified K562 cells are also used in expansion systems. K562-IL-15Rα-4-1BBL feeder cells, in combination with soluble IL-15, stimulate NK cell expansion with increased expression of natural cytotoxicity receptors, correlating with enhanced cytolytic functions (271, 272). Furthermore, K562-mbIL-21 cells can be developed to act as feeders and are reported to promote more vigorous NK cell expansion compared with K562-mbIL-15 cells (273). EBV-BLCL and autologous cells are also used as feeder cells, combined with the addition of IL-2, IL-15 and OKT3 (anti-CD3 antibody to inhibit T cell expansion) (263, 274). The protocols that expand NK cells without feeder cells are also applied for NK cell treatment. The PB mononuclear cells from healthy donors or leukemia patients are cultured in stem cell growth medium supplemented with 5% human serum with the addition of IL-2 and OKT3 (275–277). Moreover, the expanded NK cells show significant cytotoxicity toward primary leukemia cells and K562 cells. Overall, primary NK cell expansion is primarily based on the help of feeder cells to provide appropriate signals from cytokines and activating ligands and can also be expanded merely with cytokines.

### Differentiation and Expansion of NK Cells from BM or UCB CD34^+^ Cells

Initially, BM CD34^+^ cells were widely used for the generation of NK cells, but UCB CD34^+^ cells have become more frequently utilized as UCB is easier to obtain and is rich in HSCs (2, 278–282) (Figure 2). An efficient protocol to expand NK cells from CD34^+^ cells was established by the group of Spanholtz et al. (283, 284). They separated UCB CD34^+^ cells and then cultured them in a clinical-grade culture medium with human serum and a mixture of cytokines, such as FL, KL, IL-7, GM-CSF, TPO, IL-15, and IL-2, and heparin in the absence of feeder cells. They can acquire up to 10^9^ CD34^+^cell-derived NK cells in fully closed static cell culture bags and automated bioreactors that can effectively kill leukemia cells *in vitro* or *in vivo*. These NK cell products have been used in a phase I trial to treat elderly AML patients (CCMO nr. NL31699 and Dutch Trial Register nr. 2818). Other protocols that improve NK cell development and function by adding methylprednisolone to induce preferential differentiation of HSCs toward NK cells or by adding IL-12 or IL-21 to promote NK cell maturation and cytotoxicity to tumor cells are developing (189, 190, 285). The improvement of protocols to acquire functional NK cells with high quantity can be efficiently applied in the clinic.

### Improving NK Cell Survival and Function in Clinical Treatments

Natural killer cells, which are derived and expanded from autologous or allogeneic blood samples, can be applied in adoptive therapy. However, the low concentration or absence of cytokines in the body has often limited NK cell persistence postinfusion. To improve *in vivo* expansion, the Dario Campana group linked the human *IL15* gene to the gene encoding the transmembrane domain of *CD8*α (mbIL15) (131). The mbIL-15-NK cells can survive and proliferate *in vitro* or *in vivo* without exogenous cytokines. They have superior cytotoxicity against solid tumors and leukemia cells *in vitro* and against leukemia cells in xenograft models, indicating that the expression of mbIL15 may improve the postinfusion cytotoxic capacity of NK cells. Similarly, we have noted that IL-15 can induce prolonged NK cell antitumor effects after cytokine withdrawal, which suggests that IL-15 can be widely used in adoptive NK cell therapy (168). Moreover, the super-agonist IL-15-IL-15Rα-Sushi-Fc fusion protein (ALT-803) potently stimulates NK cell cytotoxic activity than native IL-15 (286) and has been used in the clinical trail to evaluate its safety and efficacy (NCT02099539). Additionally, preactivation of NK cells with IL-12/15/18 can induce memory-like NK cells with enhanced cytotoxicity toward tumors. The cells have been used in the clinical treatment with AML patients (NCT01898793). IL-12 and IL-21 can promote NK cell maturation with improved functions, which are good candidates to be applied in NK cell adoptive therapy (189, 190, 212).

Chimeric antigen receptor (CAR)-modified NK cells display a new possibility for the application of adoptive NK cell-based therapy (287). Preclinical studies to utilize CAR-expressing NK cells targeting CD19 or CD20 in B cell leukemia show effective killing toward tumor cells (267). In addition, CD19-CAR NK cells have been applied in treating B-ALL (NCT01974479) or ALL and CLL (NCT03056339) in clinical trails. To improve the efficacy of CAR-NK cells, the efforts to add genes that can elicit IL-15 production or other activating signals are now underway (288). However, new strategies still need to be developed to overcome the low transfection efficiency of NK cells.

Negative regulators can be treated as immune-checkpoints to shape immune responses. KIR and NKG2A are well-studied immune-checkpoints of NK cells, which can be blocked to gain better NK cell efficacy (289, 290) (Figure 2). The combination of anti-KIR mAbs lirilumab and lenalidomide has been used in a Phase I clinical trial (NCT01217203) with multiple myeloma patients. However, the outcomes need further study. Furthermore, anti-NKG2A antibody has also been applied in multiple clinical trials for patients with chronic lymphocytic leukemia (NCT02557516), squamous cell carcinoma of the head and neck (NCT02643550), gynecologic malignancies (NCT02459301), and squamous cell carcinoma of the oral cavity (NCT02331875). Other strategies are developing to upregulate activating signals that can significantly prolong antitumor activity of NK cells, such as retroviral transduction of NKG2D-DAP10-CD3ζ in NK cells (291) (Figure 2). The design of bi-specific antibodies that link the antigens on tumor cells, such as CD33, CD20, and CD19, together with CD16 on NK cells direct NK cells toward tumors and elicit efficient tumor cell killing(292). Additionally, the tri-specific antibody that integrates IL-15 in the existing bi-specific antibody further promotes NK cell activation to facilitate NK cell cytotoxicity (293). Overall, the developments to improve the efficacy of NK cell adoptive therapy are ongoing and may result in broader clinical applications in the near future.

## Conclusion

The development and functional maturation of NK cells are controlled by diverse cytokines. Different cytokine cocktails are needed for distinct NK cell developmental stages that are guided by the expression pattern of relative cytokine receptors. NK cells are heterogeneous and can be divided into cytotoxic, tolerant and regulatory NK cells. They distribute throughout the body in different tissues and can be shaped by their specific tissue environment via diverse combinations of cytokines. Given a robust understanding of each cytokine in NK cell development and function, NK cells can be differentiated and expanded *in vitro* to generate sufficient numbers for clinical treatment. NK cells derived from primary NK cells mainly require cytokines to promote NK cell expansion and function, such as IL-2, IL-12, and IL-15. Cells from HSC differentiation need cytokines to promote the survival and proliferation of HSCs, such as FL, KL, and IL-3, and to specify differentiation to NK cells with high cytotoxicity, such as IL-15, IL-2, IL-12, and IL-21. The application of IL-15 or IL-12/15/18 can further enhance NK cell cytotoxicity to induce greater efficacy for adoptive transfer therapy. Overall, understanding the primary roles and modes of action of each cytokine is critical to apply them more effectively in the clinic.

## Author Contributions

YW wrote the manuscript. ZT and HW designed the review and revised the manuscript.

## Conflict of Interest Statement

The authors declare that the research was conducted in the absence of any commercial or financial relationships that could be construed as a potential conflict of interest. The reviewer, MG, and handling editor declared their shared affiliation, and the handling editor states that the process nevertheless met the standards of a fair and objective review.

## References

[1] Herberman RB Nunn ME Lavrin DH Natural cytotoxic reactivity of mouse lymphoid cells against syngeneic acid allogeneic tumors. I. Distribution of reactivity and specificity. Int J Cancer (1975) 16(2):216–29. 10.1002/ijc.2910160204 50294

[2] Kiessling R Klein E Wigzell H “Natural” killer cells in the mouse. I. Cytotoxic cells with specificity for mouse Moloney leukemia cells. Specificity and distribution according to genotype. Eur J Immunol (1975) 5(2):112–7. 10.1002/eji.1830050208 1234049

[3] Vivier E Tomasello E Baratin M Walzer T Ugolini S. Functions of natural killer cells. Nat Immunol (2008) 9(5):503–10. 10.1038/ni1582 18425107

[4] Bjorkstrom NK Ljunggren HG Michaelsson J Emerging insights into natural killer cells in human peripheral tissues. Nat Rev Immunol (2016) 16(5):310–20. 10.1038/nri.2016.34 27121652

[5] Lanier LL Le AM Civin CI Loken MR Phillips JH The relationship of Cd16 (Leu-11) and Leu-19 (Nkh-1) antigen expression on human peripheral-blood NK cells and cytotoxic lymphocytes-T. J Immunol (1986) 136(12):4480–6. 3086432

[6] Cooper MA Fehniger TA Turner SC Chen KS Ghaheri BA Ghayur T Human natural killer cells: a unique innate immunoregulatory role for the CD56(bright) subset. Blood (2001) 97(10):3146–51. 10.1182/blood.V97.10.3146 11342442

[7] Fauriat C Long EO Ljunggren HG Bryceson YT Regulation of human NK-cell cytokine and chemokine production by target cell recognition. Blood (2010) 115(11):2167–76. 10.1182/blood-2009-08-238469 19965656 PMC2844017

[8] Steinert EM Schenkel JM Fraser KA Beura LK Manlove LS Igyarto BZ Quantifying memory CD8 T cells reveals regionalization of immunosurveillance. Cell (2015) 161(4):737–49. 10.1016/j.cell.2015.03.031 25957682 PMC4426972

[9] Mackay LK Rahimpour A Ma JZ Collins N Stock AT Hafon ML The developmental pathway for CD103(+)CD8(+) tissue-resident memory T cells of skin. Nat Immunol (2013) 14(12):1294–301. 10.1038/ni.2744 24162776

[10] Peng H Jiang XJ Chen YL Sojka DK Wei HM Gao X Liver-resident NK cells confer adaptive immunity in skin-contact inflammation. J Clin Invest (2013) 123(4):1444–56. 10.1172/JCI66381 23524967 PMC3613925

[11] Cortez VS Fuchs A Cella M Gilfillan S Colonna M. Cutting edge: salivary gland NK cells develop independently of Nfil3 in steady-state. J Immunol (2014) 192(10):4487–91. 10.4049/jimmunol.1303469 24740507

[12] Sojka DK Plougastel-Douglas B Yang LP Pak-Wittel MA Artyomov MN Ivanova Y Tissue-resident natural killer (NK) cells are cell lineages distinct from thymic and conventional splenic NK cells. Elife (2014) 3:e01659. 10.7554/eLife.01659 24714492 PMC3975579

[13] Marquardt N Beziat V Nystrom S Hengst J Ivarsson MA Kekalainen E Cutting edge: identification and characterization of human intrahepatic CD49a(+) NK cells. J Immunol (2015) 194(6):2467–71. 10.4049/jimmunol.1402756 25672754

[14] Peng H Wisse E Tian ZG Liver natural killer cells: subsets and roles in liver immunity. Cell Mol Immunol (2016) 13(3):328–36. 10.1038/cmi.2015.96 26639736 PMC4856807

[15] Tabiasco J Rabot M Aguerre-Girr M El Costa H Berrebi A Parant O Human decidual NK cells: unique phenotype and functional properties – a review. Placenta (2006) 27:S34–9. 10.1016/j.placenta.2006.01.009 16516963

[16] Moffett A Colucci F. Uterine NK cells: active regulators at the maternal-fetal interface. J Clin Invest (2014) 124(5):1872–9. 10.1172/JCI68107 24789879 PMC4001528

[17] Keskin DB Allan DSJ Rybalov B Andzelm MM Stern JNH Kopcow HD TGF beta promotes conversion of CD16(+) peripheral blood NK cells into CD16(-) NK cells with similarities to decidual NK cells. Proc Natl Acad Sci U S A (2007) 104(9):3378–83. 10.1073/pnas.061198104 17360654 PMC1805591

[18] Cerdeira AS Rajakumar A Royle CM Lo A Husain Z Thadhani RI Conversion of peripheral blood NK cells to a decidual NK-like phenotype by a cocktail of defined factors. J Immunol (2013) 190(8):3939–48. 10.4049/jimmunol.1202582 23487420 PMC3742368

[19] Wilkens J Male V Ghazal P Forster T Gibson DA Williams ARW Uterine NK cells regulate endometrial bleeding in women and are suppressed by the progesterone receptor modulator asoprisnil. J Immunol (2013) 191(5):2226–35. 10.4049/jimmunol.1300958 23913972 PMC3843142

[20] Eksteen B Afford SC Wigmore SJ Holt AP Adams DH. Immune-mediated liver injury. Semin Liver Dis (2007) 27(4):351–66. 10.1055/s-2007-991512 17979072

[21] Tu Z Bozorgzadeh A Pierce RH Kurtis J Crispe IN Orloff MS. TLR-dependent cross talk between human Kupffer cells and NK cells. J Exp Med (2008) 205(1):233–44. 10.1084/jem.20072195 18195076 PMC2234385

[22] Lassen MG Lukens JR Dolina JS Brown MG Hahn YS Intrahepatic IL-10 maintains NKG2A(+)Ly49(-) liver NK cells in a functionally hyporesponsive state. J Immunol (2010) 184(5):2693–701. 10.4049/jimmunol.0901362 20124099 PMC2885840

[23] Sun HY Sun C Tian ZG Xiao WH NK cells in immunotolerant organs. Cell Mol Immunol (2013) 10(3):202–12. 10.1038/cmi.2013.9 23563087 PMC4012777

[24] Kumar V Benezra J Bennett M Sonnenfeld G Natural killer cells in mice treated with Sr-89 – normal target-binding cell numbers but inability to kill even after interferon administration. J Immunol (1979) 123(4):1832–8. 383842

[25] Di Santo JP Vosshenrich CAJ. Bone marrow versus thymic pathways of natural killer cell development. Immunol Rev (2006) 214:35–46. 10.1111/j.1600-065X.2006.00461.x 17100874

[26] Vosshenrich CAJ Garcia-Ojeda ME Samson-Villeger SI Pasqualetto V Enault L Goff ORL A thymic pathway of mouse natural killer cell development characterized by expression of GATA-3 and CD127. Nat Immunol (2006) 7(11):1217–24. 10.1038/ni1395 17013389

[27] Moroso V Famili F Papazian N Cupedo T van der Laan LJW Kazemier G NK cells can generate from precursors in the adult human liver. Eur J Immunol (2011) 41(11):3340–50. 10.1002/eji.201141760 21830211

[28] Vosshenrich CAJ Samson-Villeger SI Di Santo JP. Distinguishing features of developing natural killer cells. Curr Opin Immunol (2005) 17(2):151–8. 10.1016/j.coi.2005.01.005 15766674

[29] Laurenti E Doulatov S Zandi S Plumb I Chen J April C The transcriptional architecture of early human hematopoiesis identifies multilevel control of lymphoid commitment. Nat Immunol (2013) 14(7):756–63. 10.1038/ni.2615 23708252 PMC4961471

[30] Renoux VM Zriwil A Peitzsch C Michaelsson J Friberg D Soneji S Identification of a human natural killer cell lineage-restricted progenitor in fetal and adult tissues. Immunity (2015) 43(2):394–407. 10.1016/j.immuni.2015.07.011 26287684

[31] Khaled AR Durum SK Lymphocide: cytokines and the control of lymphoid homeostasis. Nat Rev Immunol (2002) 2(11):817–30. 10.1038/Nri931 12415306

[32] Boos MD Ramirez K Kee BL Extrinsic and intrinsic regulation of early natural killer cell development. Immunol Res (2008) 40(3):193–207. 10.1007/s12026-007-8006-9 18266115

[33] Marcais A Viel S Grau M Henry T Marvel J Walzer T. Regulation of mouse NK cell development and function by cytokines. Front Immunol (2013) 4:450. 10.3389/Fimmu.2013.00450 24376448 PMC3859915

[34] Male V Brady HJM Transcriptional control of NK cell differentiation and function. Curr Top Microbiol Immunol (2014) 381:173–87. 10.1007/82_2014_376 24850220

[35] Sun JC. Transcriptional control of NK cells. Nat Killer Cells (2016) 395:1–36. 10.1007/82_2015_452 26177585

[36] Samson SI Richard O Tavian M Ranson T Vosshenrich CAJ Colucci F GATA-3 promotes maturation, IFN-gamma production, and liver-specific homing of NK cells. Immunity (2003) 19(5):701–11. 10.1016/S1074-7613(03)00294-2 14614857

[37] Townsend MJ Weinmann AS Matsuda JL Salomon R Farnham PJ Biron CA T-bet regulates the terminal maturation and homeostasis of NK and V alpha 14i NKT cells. Immunity (2004) 20(4):477–94. 10.1016/S1074-7613(04)00076-7 15084276

[38] Gascoyne DM Long E Veiga-Fernandes H de Boer J Williams O Seddon B The basic leucine zipper transcription factor E4BP4 is essential for natural killer cell development. Nat Immunol (2009) 10(10):1118–24. 10.1038/ni.1787 19749763

[39] Daussy C Faure F Mayol K Viel S Gasteiger G Charrier E T-bet and Eomes instruct the development of two distinct natural killer cell lineages in the liver and in the bone marrow. J Exp Med (2014) 211(3):563–77. 10.1084/jem.20131560 24516120 PMC3949572

[40] Gordon SM Chaix J Rupp LJ Wu JM Madera S Sun JC The transcription factors T-bet and eomes control key checkpoints of natural killer cell maturation. Immunity (2012) 36(1):55–67. 10.1016/j.immuni.2011.11.016 22261438 PMC3381976

[41] Lyman SD Jacobsen SE c-kit ligand and Flt3 ligand: stem/progenitor cell factors with overlapping yet distinct activities. Blood (1998) 91(4):1101–34. 9454740

[42] Robin C Durand C The roles of BMP and IL-3 signaling pathways in the control of hematopoietic stem cells in the mouse embryo. Int J Dev Biol (2010) 54(6–7):1189–200. 10.1387/ijdb.093040cr 20711995

[43] Lodolce JP Boone DL Chai S Swain RE Dassopoulos T Trettin S IL-15 receptor maintains lymphoid homeostasis by supporting lymphocyte homing and proliferation. Immunity (1998) 9(5):669–76. 10.1016/S1074-7613(00)80664-0 9846488

[44] Kennedy MK Glaccum M Brown SN Butz EA Viney JL Embers M Reversible defects in natural killer and memory CD8 T cell lineages in interleukin 15-deficient mice. J Exp Med (2000) 191(5):771–80. 10.1084/jem.191.5.771 10704459 PMC2195858

[45] Kundig TM Schorle H Bachmann MF Hengartner H Zinkernagel RM Horak I Immune-responses in interleukin-2 deficient mice. Science (1993) 262(5136):1059–61. 10.1126/science.8235625 8235625

[46] von Freeden-Jeffry U Vieira P Lucian LA McNeil T Burdach SE Murray R. Lymphopenia in interleukin (IL)-7 gene-deleted mice identifies IL-7 as a nonredundant cytokine. J Exp Med (1995) 181(4):1519. 10.1084/jem.181.4.1519 7699333 PMC2191954

[47] Maki K Sunaga S Komagata Y Kodaira Y Mabuchi A Karasuyama H Interleukin 7 receptor-deficient mice lack gamma delta T cells. Proc Natl Acad Sci U S A (1996) 93(14):7172–7. 10.1073/pnas.93.14.7172 8692964 PMC38955

[48] Kasaian MT Whitters MJ Carter LL Lowe LD Jussif JM Deng BJ IL-21 limits NK cell responses and promotes antigen-specific T cell activation: a mediator of the transition from innate to adaptive immunity. Immunity (2002) 16(4):559–69. 10.1016/S1074-7613(02)00295-9 11970879

[49] Lotzova E Savary CA Herberman RB Induction of NK cell-activity against fresh human-leukemia in culture with interleukin-2. J Immunol (1987) 138(8):2718–27. 3494084

[50] Biron CA Young HA Kasaian MT Interleukin-2-induced proliferation of murine natural-killer cells in vivo. J Exp Med (1990) 171(1):173–88. 10.1084/jem.171.1.173 1688606 PMC2187657

[51] Skak K Frederiksen KS Lundsgaard D. Interleukin-21 activates human natural killer cells and modulates their surface receptor expression. Immunology (2008) 123(4):575–83. 10.1111/j.1365-2567.2007.02730.x 18005035 PMC2433320

[52] Dexter TM Moore MA In vitro duplication and “cure” of haemopoietic defects in genetically anaemic mice. Nature (1977) 269(5627):412–4. 10.1038/269412a0 561894

[53] Kitamura Y Go S Decreased production of mast-cells in S1-S1d anemic mice. Blood (1979) 53(3):492–7. 367470

[54] Ullrich A Schlessinger J Signal transduction by receptors with tyrosine kinase-activity. Cell (1990) 61(2):203–12. 10.1016/0092-8674(90)90801-K 2158859

[55] Barker JE Sl/Sl(D) hematopoietic progenitors are deficient in-situ. Exp Hematol (1994) 22(2):174–7. 7507859

[56] Colucci F Di Santo JP. The receptor tyrosine kinase c-kit provides a critical signal for survival, expansion, and maturation of mouse natural killer cells. Blood (2000) 95(3):984–91. 10648413

[57] Rosnet O Marchetto S Delapeyriere O Birnbaum D Murine Flt3, a gene encoding a novel tyrosine kinase receptor of the PDGFR/CSF1R family. Oncogene (1991) 6(9):1641–50. 1656368

[58] Mackarehtschian K Hardin JD Moore KA Boast S Goff SP Lemischka IR. Targeted disruption of the flk2/flt3 gene leads to deficiencies in primitive hematopoietic progenitors. Immunity (1995) 3(1):147–61. 10.1016/1074-7613(95)90167-1 7621074

[59] McKenna HJ Stocking KL Miller RE Brasel K De Smedt T Maraskovsky E Mice lacking flt3 ligand have deficient hematopoiesis affecting hematopoietic progenitor cells, dendritic cells, and natural killer cells. Blood (2000) 95(11):3489–97. 10828034

[60] Sitnicka E Bryder D Theilgaard-Monch K Buza-Vidas N Adolfsson J Jacobsen SEW Key role of flt3 ligand in regulation of the common lymphoid progenitor but not in maintenance of the hematopoietic stem cell pool. Immunity (2002) 17(4):463–72. 10.1016/S1074-7613(02)00419-3 12387740

[61] Lantz CS Boesiger J Song CH Mach N Kobayashi T Mulligan RC Role for interleukin-3 in mast-cell and basophil development and in immunity to parasites. Nature (1998) 392(6671):90–3. 10.1038/32190 9510253

[62] Reddy EP Korapati A Chaturvedi P Rane S IL-3 signaling and the role of Src kinases, JAKs and STATs: a covert liaison unveiled. Oncogene (2000) 19(21):2532–47. 10.1038/sj.onc.1203594 10851052

[63] Robin C Ottersbach K Durand C Peeters M Vanes L Tybulewicz V An unexpected role for IL-3 in the embryonic development of hematopoietic stem cells. Dev Cell (2006) 11(2):171–80. 10.1016/j.devcel.2006.07.002 16890157

[64] Peschon JJ Morrissey PJ Grabstein KH Ramsdell FJ Maraskovsky E Gliniak BC Early lymphocyte expansion is severely impaired in interleukin-7 receptor-deficient mice. J Exp Med (1994) 180(5):1955–60. 10.1084/jem.180.5.1955 7964471 PMC2191751

[65] Palmer MJ Mahajan VS Trajman LC Irvine DJ Lauffenburger DA Chen JZ. Interleukin-7 receptor signaling network: an integrated systems perspective. Cell Mol Immunol (2008) 5(2):79–89. 10.1038/cmi.2008.10 18445337 PMC2763551

[66] Kovanen PE Leonard WJ. Cytokines and immunodeficiency diseases: critical roles of the gamma(c)-dependent cytokines interleukins 2, 4, 7, 9, 15, and 21, and their signaling pathways. Immunol Rev (2004) 202:67–83. 10.1111/j.0105-2896.2004.00203.x 15546386

[67] Sadlack B Merz H Schorle H Schimpl A Feller AC Horak I. Ulcerative colitis-like disease in mice with a disrupted interleukin-2 gene. Cell (1993) 75(2):253–61. 10.1016/0092-8674(93)80067-O 8402910

[68] Suzuki H Kundig TM Furlonger C Wakeham A Timms E Matsuyama T Deregulated T-cell activation and autoimmunity in mice lacking interleukin-2 receptor-beta. Science (1995) 268(5216):1472–6. 10.1126/science.7770771 7770771

[69] Willerford DM Chen JZ Ferry JA Davidson L Ma A Alt FW Interleukin-2 receptor-alpha chain regulates the size and content of the peripheral lymphoid compartment. Immunity (1995) 3(4):521–30. 10.1016/1074-7613(95)90180-9 7584142

[70] Sim GC Radvanyi L The IL-2 cytokine family in cancer immunotherapy. Cytokine Growth Factor Rev (2014) 25(4):377–90. 10.1016/j.cytogfr.2014.07.018 25200249

[71] Kaplan MH Sun YL Hoey T Grusby MJ. Impaired IL-12 responses and enhanced development of Th2 cells in Stat4-deficient mice. Nature (1996) 382(6587):174–7. 10.1038/382174a0 8700209

[72] Mattner F Magram J Ferrante J Launois P DiPadova K Behin R Genetically resistant mice lacking interleukin-12 are susceptible to infection with *Leishmania major* and mount a polarized Th2 cell response. Eur J Immunol (1996) 26(7):1553–9. 10.1002/eji.1830260722 8766560

[73] McIntyre KW Shuster DJ Gillooly KM Warrier RR Connaughton SE Hall LB Reduced incidence and severity of collagen-induced arthritis in interleukin-12-deficient mice. Eur J Immunol (1996) 26(12):2933–8. 10.1002/eji.1830261219 8977288

[74] Trinchieri G Pflanz S Kastelein RA The IL-12 family of heterodimeric cytokines: new players in the regulation of T cell responses. Immunity (2003) 19(5):641–4. 10.1016/S1074-7613(03)00296-6 14614851

[75] Takeda K Tsutsui H Yoshimoto T Adachi O Yoshida N Kishimoto T Defective NK cell activity and Th1 response in IL-18-deficient mice. Immunity (1998) 8(3):383–90. 10.1016/S1074-7613(00)80543-9 9529155

[76] Hoshino K Tsutsui H Kawai T Takeda K Nakanishi K Takeda Y Cutting edge: generation of IL-18 receptor-deficient mice: evidence for IL-1 receptor-related protein as an essential IL-18 binding receptor. J Immunol (1999) 162(9):5041–4. 10227969

[77] Suzuki N Chen NJ Millar DG Suzuki S Horacek T Hara H IL-1 receptor-associated kinase 4 is essential for IL-18-mediated NK and Th1 cell responses. J Immunol (2003) 170(8):4031–5. 10.4049/jimmunol.170.8.4031 12682231

[78] Lee JK Kim SH Lewis EC Azam T Reznikov LL Dinarello CA. Differences in signaling pathways by IL-1 beta and IL-18. Proc Natl Acad Sci U S A (2004) 101(23):8815–20. 10.1073/pnas.0402800101 15161979 PMC423278

[79] Ozaki K Spolski R Feng CG Qi CF Cheng J Sher A A critical role for IL-21 in regulating immunoglobulin production. Science (2002) 298(5598):1630–4. 10.1126/science.1077002 12446913

[80] Nurieva R Yang XXO Martinez G Zhang YL Panopoulos AD Ma L Essential autocrine regulation by IL-21 in the generation of inflammatory T cells. Nature (2007) 448(7152):480–8. 10.1038/nature05969 17581589

[81] Zeng R Spolski R Casas E Zhu W Levy DE Leonard WJ. The molecular basis of IL-21-mediated proliferation. Blood (2007) 109(10):4135–42. 10.1182/blood-2006-10-054973 17234735 PMC1885510

[82] Zhou LA Ivanov II Spolski R Min R Shenderov K Egawa T IL-6 programs TH-17 cell differentiation by promoting sequential engagement of the IL-21 and IL-23 pathways. Nat Immunol (2007) 8(9):967–74. 10.1038/ni1488 17581537

[83] Kuhn R Lohler J Rennick D Rajewsky K Muller W. Interleukin-10-deficient mice develop chronic enterocolitis. Cell (1993) 75(2):263–74. 10.1016/0092-8674(93)80068-P 8402911

[84] Berg DJ Davidson N Kuhn R Muller W Menon S Holland G Enterocolitis and colon cancer in interleukin-10-deficient mice are associated with aberrant cytokine production and CD4(+) TH1-like responses. J Clin Invest (1996) 98(4):1010–20. 10.1172/Jci118861 8770874 PMC507517

[85] Davidson NJ Leach MW Fort MM Thompson-Snipes L Kühn R Müller W T helper cell 1-type CD4+ T cells, but not B cells, mediate colitis in interleukin 10-deficient mice. J Exp Med (1996) 184(1):241. 10.1084/jem.184.1.241 8691138 PMC2192682

[86] Moore KW Malefyt RD Coffman RL O’Garra A Interleukin-10 and the interleukin-10 receptor. Annu Rev Immunol (2001) 19:683–765. 10.1146/annurev.immunol.19.1.683 11244051

[87] Shull MM Ormsby I Kier AB Pawlowski S Diebold RJ Yin MY Targeted disruption of the mouse transforming growth factor-beta-1 gene results in multifocal inflammatory disease. Nature (1992) 359(6397):693–9. 10.1038/359693a0 1436033 PMC3889166

[88] Kulkarni AB Huh CG Becker D Geiser A Lyght M Flanders KC Transforming growth factor-beta-1 null mutation in mice causes excessive inflammatory response and early death. Proc Natl Acad Sci U S A (1993) 90(2):770–4. 10.1073/pnas.90.2.770 8421714 PMC45747

[89] Gorelik L Flavell RA Abrogation of TGF beta signaling in T cells leads to spontaneous T cell differentiation and autoimmune disease. Immunity (2000) 12(2):171–81. 10.1016/S1074-7613(00)80170-3 10714683

[90] Shi YG Massague J Mechanisms of TGF-beta signaling from cell membrane to the nucleus. Cell (2003) 113(6):685–700. 10.1016/S0092-8674(03)00432-X 12809600

[91] Artis D Spits H The biology of innate lymphoid cells. Nature (2015) 517(7534):293–301. 10.1038/nature14189 25592534

[92] Freud AG Yokohama A Becknell B Lee MT Mao HC Ferketich AK Evidence for discrete stages of human natural killer cell differentiation in vivo. J Exp Med (2006) 203(4):1033–43. 10.1084/jem.20052507 16606675 PMC2118285

[93] Sawai CM Babovic S Upadhaya S Knapp DJHF Lavin Y Lau CM Hematopoietic stem cells are the major source of multilineage hematopoiesis in adult animals. Immunity (2016) 45(3):597–609. 10.1016/j.immuni.2016.08.007 27590115 PMC5054720

[94] Galli SJ Zsebo KM Geissler EN The kit-ligand, stem-cell factor. Adv Immunol (1994) 55:1–96. 10.1016/S0065-2776(08)60508-8 7508174

[95] Broudy VC Stem cell factor and hematopoiesis. Blood (1997) 90(4):1345–64. 9269751

[96] Flanagan JG Chan DC Leder P Transmembrane form of the kit ligand growth-factor is determined by alternative splicing and is missing in the Si(D) mutant. Cell (1991) 64(5):1025–35. 10.1016/0092-8674(91)90326-T 1705866

[97] Toksoz D Zsebo KM Smith KA Hu S Brankow D Suggs SV Support of human hematopoiesis in long-term bone-marrow cultures by murine stromal cells selectively expressing the membrane-bound and secreted forms of the human homolog of the steel gene-product, stem-cell factor. Proc Natl Acad Sci U S A (1992) 89(16):7350–4. 10.1073/pnas.89.16.7350 1380155 PMC49707

[98] Waskow C Paul S Haller C Gassmann M Rodewald HR Viable c-Kit(W/W) mutants reveal pivotal role for c-kit in the maintenance of lymphopoiesis. Immunity (2002) 17(3):277–88. 10.1016/S1074-7613(02)00386-2 12354381

[99] Gwin KA Shapiro MB Dolence JJ Huang ZXL Medina KL. Hoxa9 and Flt3 signaling synergistically regulate an early checkpoint in lymphopoiesis. J Immunol (2013) 191(2):745–54. 10.4049/jimmunol.1203294 23772038 PMC3702669

[100] Yu HX Fehniger TA Fuchshuber P Thiel KS Vivier E Carson WE Flt3 ligand promotes the generation of a distinct CD34(+) human natural killer cell progenitor that responds to interleukin-15. Blood (1998) 92(10):3647–57. 9808558

[101] Broughton SE Dhagat U Hercus TR Nero TL Grimbaldeston MA Bonder CS The GM-CSF/IL-3/IL-5 cytokine receptor family: from ligand recognition to initiation of signaling. Immunol Rev (2012) 250:277–302. 10.1111/j.1600-065X.2012.01164.x 23046136

[102] Cai ZL de Bruijn M Ma XQ Dortland B Luteijn T Downing JR Haploinsufficiency of AML1 affects the temporal and spatial generation of hematopoietic stem cells in the mouse embryo. Immunity (2000) 13(4):423–31. 10.1016/S1074-7613(00)00042-X 11070161

[103] Miyajima A Mui ALF Ogorochi T Sakamaki K Receptors for granulocyte-macrophage colony-stimulating factor, interleukin-3, and interleukin-5. Blood (1993) 82(7):1960–74. 8400249

[104] Ihle JN Cytokine receptor signaling. Nature (1995) 377(6550):591–4. 10.1038/377591a0 7566171

[105] Miller JS McCullar V Verfaillie CM Ex vivo culture of CD34(+)/Lin(-)/DR- cells in stroma-derived soluble factors, interleukin-3, and macrophage inflammatory protein-1 alpha maintains not only myeloid but also lymphoid progenitors in a novel switch culture assay. Blood (1998) 91(12):4516–22. 9616147

[106] Miller JS McCullar V Punzel M Lemischka IR Moore KA Single adult human CD34(+)/Lin(-)/CD38(-) progenitors give rise to natural killer cells, B-lineage cells, dendritic cells, and myeloid cells. Blood (1999) 93(1):96–106. 9864151

[107] Muench MO Humeau L Paek B Ohkubo T Lanier LL Albanese CT Differential effects of interleukin-3, interleukin-7, interleukin 15, and granulocyte-macrophage colony-stimulating factor in the generation of natural killer and B cells from primitive human fetal liver progenitors. Exp Hematol (2000) 28(8):961–73. 10.1016/S0301-472x(00)00490-2 10989197

[108] Ficara F Superchi DB Hernandez RJ Mocchetti C Carballido-Perrig N Andolfi G IL-3 or IL-7 increases ex vivo gene transfer efficiency in ADA-SCID BM CD34(+) cells while maintaining in vivo lymphoid potential. Mol Ther (2004) 10(6):1096–108. 10.1016/j.ymthe.2004.08.014 15564141

[109] Greenbaum A Hsu YMS Day RB Schuettpelz LG Christopher MJ Borgerding JN CXCL12 in early mesenchymal progenitors is required for haematopoietic stem-cell maintenance. Nature (2013) 495(7440):227–30. 10.1038/nature11926 23434756 PMC3600148

[110] Kunisaki Y Bruns I Scheiermann C Ahmed J Pinho S Zhang DC Arteriolar niches maintain haematopoietic stem cell quiescence. Nature (2013) 502(7473):637–43. 10.1038/nature12612 24107994 PMC3821873

[111] Gomes AC Hara T Lim VY Herndler-Brandstetter D Nevius E Sugiyama T Hematopoietic stem cell niches produce lineage-instructive signals to control multipotent progenitor differentiation. Immunity (2016) 45(6):1219–31. 10.1016/j.immuni.2016.11.004 27913094 PMC5538583

[112] Mazzucchelli R Durum SK. Interleukin-7 receptor expression: intelligent design. Nat Rev Immunol (2007) 7(2):144–54. 10.1038/nri2023 17259970

[113] Takada K Jameson SC. Naive T cell homeostasis: from awareness of space to a sense of place. Nat Rev Immunol (2009) 9(12):823–32. 10.1038/nri2657 19935802

[114] Leonard WJ. Cytokines and immunodeficiency diseases. Nat Rev Immunol (2001) 1(3):200–8. 10.1038/35105066 11905829

[115] Buckley RH. Molecular defects in human severe combined immunodeficiency and approaches to immune reconstitution. Annu Rev Immunol (2004) 22:625–55. 10.1146/annurev.immunol.22.012703.104614 15032591

[116] Cooper MA Bush JE Fehniger TA VanDeusen JB Waite RE Liu Y In vivo evidence for a dependence on interleukin 15 for survival of natural killer cells. Blood (2002) 100(10):3633–8. 10.1182/blood-2001-12-0293 12393617

[117] Koka R Burkett PR Chien M Chai S Chan F Lodolce JP Interleukin (IL)-15Rα-deficient natural killer cells survive in normal but not IL-15Rα-deficient mice. J Exp Med (2003) 197(8):977 10.1084/jem.20021836 12695489 PMC2193874

[118] Zhang XH Sun SQ Hwang IK Tough DF Sprent J Potent and selective stimulation of memory-phenotype CD8(+) T cells in vivo by IL-15. Immunity (1998) 8(5):591–9. 10.1016/S1074-7613(00)80564-6 9620680

[119] Ku CC Murakami M Sakamato A Kappler J Marrack P Control of homeostasis of CD8(+) memory T cells by opposing cytokines. Science (2000) 288(5466):675–8. 10.1126/science.288.5466.675 10784451

[120] Matsuda JL Gapin L Sidobre S Kieper WC Tan JYT Ceredig R Homeostasis of V(alpha)14i NKT cells. Nat Immunol (2002) 3(10):966–74. 10.1038/ni837 12244311

[121] Becknell B Caligiuri MA. Interleukin-2, interleukin-15, and their roles in human natural killer cells. Adv Immunol (2005) 86:209–39. 10.1016/S0065-2776(04)86006-1 15705423

[122] Giri JG Ahdieh M Eisenman J Shanebeck K Grabstein K Kumaki S Utilization of the beta-chain and gamma-chain of the Il-2 receptor by the novel cytokine-Il-15. EMBO J (1994) 13(12):2822–30. 8026467 10.1002/j.1460-2075.1994.tb06576.x PMC395163

[123] Grabstein KH Eisenman J Shanebeck K Rauch C Srinivasan S Fung V Cloning of a T-cell growth-factor that interacts with the beta-chain of the interleukin-2 receptor. Science (1994) 264(5161):965–8. 10.1126/science.8178155 8178155

[124] Miller JS Alley KA Mcglave P Differentiation of natural-killer (Nk) cells from human primitive marrow progenitors in a stroma-based long-term culture system – identification of a Cd34(+)7(+) NK progenitor. Blood (1994) 83(9):2594–601. 7513206

[125] Freud AG Becknell B Roychowdhury S Mao HC Ferketich AK Nuovo GJ A human CD34(+) subset resides in lymph nodes and differentiates into CD56(bright) natural killer cells. Immunity (2005) 22(3):295–304. 10.1016/j.immuni.2005.01.013 15780987

[126] Dubois S Mariner J Waldmann TA Tagaya Y. IL-15R alpha recycles and presents IL-15 in trans to neighboring cells. Immunity (2002) 17(5):537–47. 10.1016/S1074-7613(02)00429-6 12433361

[127] Burkett PR Koka R Chien M Chai S Boone DL Ma A Coordinate expression and trans presentation of interleukin (IL)-15R alpha and IL-15 supports natural killer cell and memory CD8(+) T cell homeostasis. J Exp Med (2004) 200(7):825–34. 10.1084/jem.20041389 15452177 PMC2213280

[128] Mortier E Woo T Advincula R Gozalo S Ma A. IL-15R alpha chaperones IL-15 to stable dendritic cell membrane complexes that activate NK cells via trans presentation. J Exp Med (2008) 205(5):1213–25. 10.1084/jem.20071913 18458113 PMC2373851

[129] Huntington ND Legrand N Alves NL Jaron B Weijer K Plet A IL-15 trans-presentation promotes human NK cell development and differentiation in vivo. J Exp Med (2009) 206(1):25–34. 10.1084/jem.20082013 19103877 PMC2626663

[130] Bobbala D Mayhue M Menendez A Ilangumaran S Ramanathan S Trans-presentation of interleukin-15 by interleukin-15 receptor alpha is dispensable for the pathogenesis of autoimmune type 1 diabetes. Cell Mol Immunol (2017) 14(7):590–6. 10.1038/cmi.2015.102 26853723 PMC5520411

[131] Imamura M Shook D Kamiya T Shimasaki N Chai SMH Coustan-Smith E Autonomous growth and increased cytotoxicity of natural killer cells expressing membrane-bound interleukin-15. Blood (2014) 124(7):1081–8. 10.1182/blood-2014-02-556837 25006133

[132] Ali AK Nandagopal N Lee SH. IL-15-PI3K-AKT-mTOR: a critical pathway in the life journey of natural killer cells. Front Immunol (2015) 6:355. 10.3389/Fimmu.2015.00355 26257729 PMC4507451

[133] Imada K Bloom ET Nakajima H Horvath-Arcidiacono JA Udy GB Davey HW Stat5b is essential for natural killer cell-mediated proliferation and cytolytic activity. J Exp Med (1998) 188(11):2067–74. 10.1084/jem.188.11.2067 9841920 PMC2212377

[134] Moriggl R Topham DJ Teglund S Sexl V McKay C Wang D Stat5 is required for IL-2-induced cell cycle progression of peripheral T cells. Immunity (1999) 10(2):249–59. 10.1016/S1074-7613(00)80025-4 10072077

[135] Eckelhart E Warsch W Zebedin E Simma O Stoiber D Kolbe T A novel Ncr1-Cre mouse reveals the essential role of STAT5 for NK-cell survival and development. Blood (2011) 117(5):1565–73. 10.1182/blood-2010-06-291633 21127177

[136] Bernasconi A Marino R Ribas A Rossi J Ciaccio M Oleastro M Characterization of immunodeficiency in a patient with growth hormone insensitivity secondary to a novel STAT5b gene mutation. Pediatrics (2006) 118(5):e1584–92. 10.1542/peds.2005-2882 17030597

[137] Yang MX Li D Chang Z Yang ZZ Tian ZG Dong ZJ. PDK1 orchestrates early NK cell development through induction of E4BP4 expression and maintenance of IL-15 responsiveness. J Exp Med (2015) 212(2):253–65. 10.1084/jem.20141703 25624444 PMC4322053

[138] Yokota Y Mansouri A Mori S Sugawara S Adachi S Nishikawa S Development of peripheral lymphoid organs and natural killer cells depends on the helix-loop-helix inhibitor Id2. Nature (1999) 397(6721):702–6. 10.1038/17812 10067894

[139] Boos MD Yokota Y Eberl G Kee BL. Mature natural killer cell and lymphoid tissue-inducing cell development requires Id2-mediated suppression of E protein activity. J Exp Med (2007) 204(5):1119–30. 10.1084/jem.20061959 17452521 PMC2118569

[140] Delconte RB Shi W Sathe P Ushiki T Seillet C Minnich M The helix-loop-helix protein ID2 governs NK cell fate by tuning their sensitivity to interleukin-15. Immunity (2016) 44(1):103–15. 10.1016/j.immuni.2015.12.007 26795246

[141] Fu BQ Tian ZG Wei HM. Subsets of human natural killer cells and their regulatory effects. Immunology (2014) 141(4):483–9. 10.1111/imm.12224 24303897 PMC3956422

[142] Fehniger TA Cooper MA Nuovo GJ Cella M Facchetti F Colonna M CD56(bright) natural killer cells are present in human lymph nodes and are activated by T cell-derived IL-2: a potential new link between adaptive and innate immunity. Blood (2003) 101(8):3052–7. 10.1182/blood-2002-09-2876 12480696

[143] Walzer T Dalod M Robbins SH Zitvogel L Vivier E Natural-killer cells and dendritic cells: “l’ union fait la force”. Blood (2005) 106(7):2252–8. 10.1182/blood-2005-03-1154 15933055

[144] Long EO. Ready for prime time: NK cell priming by dendritic cells. Immunity (2007) 26(4):385–7. 10.1016/j.immuni.2007.04.001 17459805

[145] Long EO Kim HS Liu D Peterson ME Rajagopalan S. Controlling natural killer cell responses: integration of signals for activation and inhibition. Annu Rev Immunol (2013) 31:227–58. 10.1146/annurev-immunol-020711-075005 23516982 PMC3868343

[146] Matek TR. The biology of interleukin-2. Annu Rev Immunol (2008) 26:453–79. 10.1146/annurev.immunol.26.021607.090357 18062768

[147] Trinchieri G Biology of natural-killer cells. Adv Immunol (1989) 47:187–376. 10.1016/S0065-2776(08)60664-1 2683611 PMC7131425

[148] Minagawa M Watanabe H Miyaji C Tomiyama K Shimura H Ito A Enforced expression of Bcl-2 restores the number of NK cells, but does not rescue the impaired development of NKT cells or intraepithelial lymphocytes, in IL-2/IL-15 receptor beta-chain-deficient mice. J Immunol (2002) 169(8):4153–60. 10.4049/jimmunol.169.8.4153 12370344

[149] Wang HM Smith KA The interleukin-2 receptor – functional consequences of its bimolecular structure. J Exp Med (1987) 166(4):1055–69. 10.1084/jem.166.4.1055 3116143 PMC2188729

[150] Takeshita T Asao H Ohtani K Ishii N Kumaki S Tanaka N Cloning of the gamma-chain of the human Il-2 receptor. Science (1992) 257(5068):379–82. 10.1126/science.1631559 1631559

[151] Rickert M Wang XQ Boulanger MJ Goriatcheva N Garcia KC. The structure of interleukin-2 complexed with its alpha receptor. Science (2005) 308(5727):1477–80. 10.1126/science.1109745 15933202

[152] Levin AM Bates DL Ring AM Krieg C Lin JT Su L Exploiting a natural conformational switch to engineer an interleukin-2 ‘superkine’. Nature (2012) 484(7395):529–33. 10.1038/nature10975 22446627 PMC3338870

[153] Ni J Miller M Stojanovic A Garbi N Cerwenka A. Sustained effector function of IL-12/15/18-preactivated NK cells against established tumors. J Exp Med (2012) 209(13):2351–65. 10.1084/jem.20120944 23209317 PMC3526364

[154] Leong JW Chase JM Romee R Schneider SE Sullivan RP Cooper MA Preactivation with IL-12, IL-15, and IL-18 induces CD25 and a functional high-affinity IL-2 receptor on human cytokine-induced memory-like natural killer cells. Biol Blood Marrow Transplant (2014) 20(4):463–73. 10.1016/j.bbmt.2014.01.006 24434782 PMC3959288

[155] Sitrin J Ring A Garcia KC Benoist C Mathis D. Regulatory T cells control NK cells in an insulitic lesion by depriving them of IL-2. J Exp Med (2013) 210(6):1153–65. 10.1084/jem.20122248 23650440 PMC3674700

[156] Martin JF Perry JSA Jakhete NR Wang X Bielekova B. An IL-2 paradox: blocking CD25 on T cells induces IL-2-driven activation of CD56(bright) NK cells. J Immunol (2010) 185(2):1311–20. 10.4049/jimmunol.0902238 20543101 PMC3085179

[157] Sim GC Liu C Wang E Liu H Creasy C Dai Z IL-2 variant circumvents ICOS+ regulatory T cell expansion and promotes NK cell activation. Cancer Immunol Res (2016) 4(11):983–94. 10.1158/2326-6066.CIR-15-0195 27697858

[158] Burton JD Bamford RN Peters C Grant AJ Kurys G Goldman CK A lymphokine, provisionally designated interleukin-T and produced by a human adult T-cell leukemia line, stimulates T-cell proliferation and the induction of lymphokine-activated killer-cells. Proc Natl Acad Sci U S A (1994) 91(11):4935–9. 10.1073/pnas.91.11.4935 8197160 PMC43904

[159] Carson WE Giri JG Lindemann MJ Linett ML Ahdieh M Paxton R Interleukin (Il)-15 is a novel cytokine that activates human natural-killer-cells via components of the Il-2 receptor. J Exp Med (1994) 180(4):1395–403. 10.1084/jem.180.4.1395 7523571 PMC2191697

[160] Ma A Koka R Burkett P Diverse functions of IL-2, IL-15, and IL-7 in lymphoid homeostasis. Annu Rev Immunol (2006) 24:657–79. 10.1146/annurev.immunol.24.021605.090727 16551262

[161] Bamford RN Grant AJ Burton JD Peters C Kurys G Goldman CK The interleukin (Il)-2 receptor-beta chain is shared by Il-2 and a cytokine, provisionally designated Il-T, that stimulates T-cell proliferation and the induction of lymphokine-activated killer-cells. Proc Natl Acad Sci U S A (1994) 91(11):4940–4. 10.1073/pnas.91.11.4940 8197161 PMC43905

[162] Giri JG Kumaki S Ahdieh M Friend DJ Loomis A Shanebeck K Identification and cloning of a novel Il-15 binding-protein that is structurally related to the alpha-chain of the Il-2 receptor. EMBO J (1995) 14(15):3654–63. 7641685 10.1002/j.1460-2075.1995.tb00035.x PMC394440

[163] Fehniger TA Shah MH Turner MJ VanDeusen JB Whitman SP Cooper MA Differential cytokine and chemokine gene expression by human NK cells following activation with IL-18 or IL-15 in combination with IL-12: implications for the innate immune response. J Immunol (1999) 162(8):4511–20. 10201989

[164] Lucas M Schachterle W Oberle K Aichele P Diefenbach A. Dendritic cells prime natural killer cells by trans-presenting interleukin 15. Immunity (2007) 26(4):503–17. 10.1016/j.immuni.2007.03.006 17398124 PMC2084390

[165] Nandagopal N Ali AK Komal AK Lee SH. The critical role of IL-15-PI3K-mTOR pathway in natural killer cell effector functions. Front Immunol (2014) 5:187. 10.3389/Fimmu.2014.00187 24795729 PMC4005952

[166] Fehniger TA Cai SF Cao X Bredemeyer AJ Presti RI French AR Acquisition of murine NK cell cytotoxicity requires the translation of a pre-existing pool of granzyme B and perforin mRNAs. Immunity (2007) 26(6):798–811. 10.1016/j.immuni.2007.04.010 17540585

[167] Marais A Cherfils-Vicini J Viant C Degouve S Viel S Fenis A The metabolic checkpoint kinase mTOR is essential for IL-15 signaling during the development and activation of NK cells. Nat Immunol (2014) 15(8):749–57. 10.1038/ni.2936 24973821 PMC4110708

[168] Mao YM van Hoef V Zhang XN Wennerberg E Lorent J Witt K IL-15 activates mTOR and primes stress-activated gene expression leading to prolonged antitumor capacity of NK cells. Blood (2016) 128(11):1475–89. 10.1182/blood-2016-02-698027 27465917 PMC5025899

[169] Kobayashi M Fitz L Ryan M Hewick RM Clark SC Chan S Identification and purification of natural-killer cell stimulatory factor (Nksf), a cytokine with multiple biologic effects on human-lymphocytes. J Exp Med (1989) 170(3):827–45. 10.1084/jem.170.3.827 2504877 PMC2189443

[170] Jalah R Rosati M Ganneru B Pilkington GR Valentin A Kulkarni V The p40 subunit of interleukin (IL)-12 promotes stabilization and export of the p35 subunit implications for improved IL-12 cytokine production. J Biol Chem (2013) 288(9):6763–76. 10.1074/jbc.M112.436675 23297419 PMC3585113

[171] Hsieh CS Macatonia SE Tripp CS Wolf SF Ogarra A Murphy KM Development of Th1 Cd4+ T-cells through Il-12 produced by *Listeria*-induced macrophages. Science (1993) 260(5107):547–9. 10.1126/science.8097338 8097338

[172] Macatonia SE Hosken NA Litton M Vieira P Hsieh CS Culpepper JA Dendritic cells produce Il-12 and direct the development of Th1 cells from naive Cd4(+) T-cells. J Immunol (1995) 154(10):5071–9. 7730613

[173] Cella M Scheidegger D PalmerLehmann K Lane P Lanzavecchia A Alber G Ligation of CD40 on dendritic cells triggers production of high levels of interleukin-12 and enhances T cell stimulatory capacity: T-T help via APC activation. J Exp Med (1996) 184(2):747–52. 10.1084/jem.184.2.747 8760829 PMC2192696

[174] Hunter CA. New IL-12-family members: IL-23 and IL-27, cytokines with divergent functions. Nat Rev Immunol (2005) 5(7):521–31. 10.1038/nri1648 15999093

[175] O’Leary JG Goodarzi M Drayton DL von Andrian UH. T cell- and B cell-independent adaptive immunity mediated by natural killer cells. Nat Immunol (2006) 7(5):507–16. 10.1038/ni1332 16617337

[176] Sun JC Madera S Bezman NA Beilke JN Kaplan MH Lanier LL Proinflammatory cytokine signaling required for the generation of natural killer cell memory. J Exp Med (2012) 209(5):947–54. 10.1084/jem.20111760 22493516 PMC3348098

[177] Paust S Gill HS Wang BZ Flynn MP Moseman EA Senman B Critical role for the chemokine receptor CXCR6 in NK cell-mediated antigen-specific memory of haptens and viruses. Nat Immunol (2010) 11(12):1127–35. 10.1038/ni.1953 20972432 PMC2982944

[178] Sun JC Beilke JN Lanier LL. Immune memory redefined: characterizing the longevity of natural killer cells. Immunol Rev (2010) 236:83–94. 10.1111/j.1600-065X.2010.00900.x 20636810 PMC2907527

[179] Vivier E Raulet DH Moretta A Caligiuri MA Zitvogel L Lanier LL Innate or adaptive immunity? The example of natural killer cells. Science (2011) 331(6013):44–9. 10.1126/science.1198687 21212348 PMC3089969

[180] Cooper MA Elliott JM Keyel PA Yang LP Carrero JA Yokoyama WM Cytokine-induced memory-like natural killer cells. Proc Natl Acad Sci U S A (2009) 106(6):1915–9. 10.1073/pnas.0813192106 19181844 PMC2644138

[181] Romee R Schneider SE Leong JW Chase JM Keppel CR Sullivan RP Cytokine activation induces human memory-like NK cells. Blood (2012) 120(24):4751–60. 10.1182/blood-2012-04-419283 22983442 PMC3520618

[182] Romee R Rosario M Berrien-Elliott MM Wagner JA Jewell BA Schappe T Cytokine-induced memory-like natural killer cells exhibit enhanced responses against myeloid leukemia. Sci Transl Med (2016) 8(357): 357ra123 10.1126/scitranslmed.aaf2341 PMC5436500 27655849

[183] Kim S Poursine-Laurent J Truscott SM Lybarger L Song YJ Yang LP Licensing of natural killer cells by host major histocompatibility complex class I molecules. Nature (2005) 436(7051):709–13. 10.1038/nature03847 16079848

[184] Anfossi N Andre P Guia S Falk CS Roetynck S Stewart CA Human NK cell education by inhibitory receptors for MHC class I. Immunity (2006) 25(2):331–42. 10.1016/j.immuni.2006.06.013 16901727

[185] Wagner JA Berrien-Elliott MM Rosario M Leong JW Jewell BA Schappe T Cytokine-induced memory-like differentiation enhances unlicensed natural killer cell antileukemia and Fc gamma RIIIa-triggered responses. Biol Blood Marrow Transplant (2017) 23(3):398–404. 10.1016/j.bbmt.2016.11.018 27894857 PMC5408734

[186] Cooley S Xiao F Pitt M Gleason M McCullar V Bergemann TL A subpopulation of human peripheral blood NK cells that lacks inhibitory receptors for self-MHC is developmentally immature. Blood (2007) 110(2):578–86. 10.1182/blood-2006-07-036228 17392508 PMC1924487

[187] Romagnani C Juelke K Falco M Morandi B D’Agostino A Costa R CD56(bright) CD16(-) killer Ig-like receptor(-) NK cells display longer telomeres and acquire features of CD56(dim) NK cells upon activation. J Immunol (2007) 178(8):4947–55. 10.4049/jimmunol.178.8.4947 17404276

[188] Juelke K Killig M Thiel A Dong J Romagnani C. Education of hyporesponsive NK cells by cytokines. Eur J Immunol (2009) 39(9):2548–55. 10.1002/eji.200939307 19701893

[189] Lehmann D Spanholtz J Sturtzel C Tordoir M Schlechta B Groenewegen D IL-12 directs further maturation of ex vivo differentiated NK cells with improved therapeutic potential. PLoS One (2014) 9(1):e87131. 10.1371/journal.pone.0087131 24498025 PMC3909052

[190] Cany J van der Waart AB Spanholtz J Tordoir M Jansen JH van der Voort R Combined IL-15 and IL-12 drives the generation of CD34(+)-derived natural killer cells with superior maturation and alloreactivity potential following adoptive transfer. Oncoimmunology (2015) 4(7):e1017701 10.1080/2162402X.2015.1017701 26140247 PMC4485802

[191] Okamura H Tsutsui H Komatsu T Yutsudo M Hakura A Tanimoto T Cloning of a new cytokine that induces IFN-gamma production by T-cells. Nature (1995) 378(6552):88–91. 10.1038/378088a0 7477296

[192] Sims JE Smith DE. The IL-1 family: regulators of immunity. Nat Rev Immunol (2010) 10(2):89–102. 10.1038/nri2691 20081871

[193] Sporri R Joller N Hilbi H Oxenius A. A novel role for neutrophils as critical activators of NK cells. J Immunol (2008) 181(10):7121–30. 10.4049/jimmunol.181.10.7121 18981133

[194] Kastenmuller W Torabi-Parizi P Subramanian N Lammermann T Germain RN. A spatially-organized multicellular innate immune response in lymph nodes limits systemic pathogen spread. Cell (2012) 150(6):1235–48. 10.1016/j.cell.2012.07.021 22980983 PMC3514884

[195] Oertli M Sundquist M Hitzler I Engler DB Arnold IC Reuter S DC-derived IL-18 drives Treg differentiation, murine *Helicobacter pylori*-specific immune tolerance, and asthma protection. J Clin Invest (2012) 122(3):1082–96. 10.1172/JCI61029 22307326 PMC3287234

[196] Pizarro TT Michie MH Bentz M Woraratanadharm J Smith MF Foley E IL-18, a novel immunoregulatory cytokine, is up-regulated in Crohn’s disease: expression and localization in intestinal mucosal cells. J Immunol (1999) 162(11):6829–35. 10352304

[197] Ghayur T Banerjee S Hugunin M Butler D Herzog L Carter A Caspase-1 processes IFN-gamma-inducing factor and regulates LPS-induced IFN-gamma production. Nature (1997) 386(6625):619–23. 10.1038/386619a0 9121587

[198] Gu Y Kuida K Tsutsui H Ku G Hsiao K Fleming MA Activation of interferon-gamma inducing factor mediated by interleukin-1 beta converting enzyme. Science (1997) 275(5297):206–9. 10.1126/science.275.5297.206 8999548

[199] Boraschi D Tagliabue A. The interleukin-1 receptor family. Semin Immunol (2013) 25(6):394–407. 10.1016/j.smim.2013.10.023 24246227

[200] Kawakami K Koguchi Y Qureshi MH Miyazato A Yara S Kinjo Y IL-18 contributes to host resistance against infection with *Cryptococcus neoformans* in mice with defective IL-12 synthesis through induction of IFN-gamma production by NK cells. J Immunol (2000) 165(2):941–7. 10.4049/jimmunol.165.2.941 10878369

[201] Pien GC Satoskar AR Takeda K Akira S Biron CA. Cutting edge: selective IL-18 requirements for induction of compartmental IFN-gamma responses during viral infection. J Immunol (2000) 165(9):4787–91. 10.4049/jimmunol.165.9.4787 11046000

[202] Rowland CA Lertmemongkolchai G Bancroft A Haque A Lever MS Griffin KF Critical role of type 1 cytokines in controlling initial infection with *Burkholderia mallei*. Infect Immun (2006) 74(9):5333–40. 10.1128/Iai.02046-05 16926428 PMC1594859

[203] Haeberlein S Sebald H Bogdan C Schleicher U. IL-18, but not IL-15, contributes to the IL-12-dependent induction of NK-cell effector functions by *Leishmania infantum* in vivo. Eur J Immunol (2010) 40(6):1708–17. 10.1002/eji.200939988 20213736 PMC2909391

[204] Stegmann KA De Souza JB Riley EM IL-18-induced expression of high-affinity IL-2R on murine NK cells is essential for NK-cell IFN-gamma production during murine *Plasmodium yoelii* infection. Eur J Immunol (2015) 45(12):3431–40. 10.1002/eji.201546018 26420375 PMC4982096

[205] Nielsen CM Wolf AS Goodier MR Riley EM Synergy between common gamma chain family cytokines and IL-18 potentiates innate and adaptive pathways of NK cell activation. Front Immunol (2016) 7:101 10.3389/Fimmu.2016.00101 27047490 PMC4801862

[206] Yoshimoto T Takeda K Tanaka T Ohkusu K Kashiwamura S Okamura H IL-12 up-regulates IL-18 receptor expression on T cells, Th1 cells, and B cells: synergism with IL-18 for IFN-gamma production. J Immunol (1998) 161(7):3400–7. 9759857

[207] Ardolino M Azimi CS Iannello A Trevino TN Horan L Zhang L Cytokine therapy reverses NK cell anergy in MHC-deficient tumors. J Clin Invest (2014) 124(11):4781–94. 10.1172/JCI74337 25329698 PMC4347250

[208] Spolski R Leonard WJ. Interleukin-21: basic biology and implications for cancer and autoimmunity. Annu Rev Immunol (2008) 26:57–79. 10.1146/annurev.immunol.26.021607.090316 17953510

[209] Ozaki K Kikly K Michalovich D Young PR Leonard WJ Cloning of a type I cytokine receptor most related to the IL-2 receptor beta chain. Proc Natl Acad Sci U S A (2000) 97(21):11439–44. 10.1073/pnas.200360997 11016959 PMC17218

[210] Parrish-Novak J Dillon SR Nelson A Hammond A Sprecher C Gross JA Interleukin 21 and its receptor are involved in NK cell expansion and regulation of lymphocyte function. Nature (2000) 408(6808):57–63. 10.1038/35040504 11081504

[211] Korn T Bettelli E Gao W Awasthi A Jager A Strom TB IL-21 initiates an alternative pathway to induce proinflammatory T(H)17 cells. Nature (2007) 448(7152):484–7. 10.1038/nature05970 17581588 PMC3805028

[212] Sivori S Cantoni C Parolini S Marcenaro E Conte R Moretta L IL-21 induces both rapid maturation of human CD34(+) cell precursors towards NK cells and acquisition of surface killer Ig-like receptors. Eur J Immunol (2003) 33(12):3439–47. 10.1002/eji.200324533 14635054

[213] Brady J Hayakawa Y Smyth MJ Nutt SL. IL-21 induces the functional maturation of murine NK cells. J Immunol (2004) 172(4):2048–58. 10.4049/jimmunol.172.4.2048 14764669

[214] Roda JM Parihar R Lehman A Mani A Tridandapani S Carson WE III. Interleukin-21 enhances NK cell activation in response to antibody-coated targets. J Immunol (2006) 177(1):120–9. 10.4049/jimmunol.177.1.120 16785506

[215] O’Connor JC McCusker RH Strle K Johnson RW Dantzer R Kelley KW. Regulation of IGF-I function by proinflammatory cytokines: at the interface of immunology and endocrinology. Cell Immunol (2008) 252(1–2):91–110. 10.1016/j.cellimm.2007.09.010 18325486 PMC2615236

[216] Ni F Sun R Fu BQ Wang FY Guo C Tian ZG IGF-1 promotes the development and cytotoxic activity of human NK cells. Nat Commun (2013) 4:1479 10.1038/ncomms2484 23403580 PMC3586714

[217] Jung HJ Suh Y Regulation of IGF-1 signaling by microRNAs. Front Genet (2014) 5:472 10.3389/fgene.2014.00472 25628647 PMC4292735

[218] Deng YC Kerdiles Y Chu JH Yuan SZ Wang YW Chen XL Transcription factor Foxo1 is a negative regulator of natural killer cell maturation and function. Immunity (2015) 42(3):457–70. 10.1016/j.immuni.2015.02.006 25769609 PMC4400836

[219] Savai R Al-Tamari HM Sedding D Kojonazarov B Muecke C Teske R Pro-proliferative and inflammatory signaling converge on FoxO1 transcription factor in pulmonary hypertension. Nat Med (2014) 20(11):1289–300. 10.1038/nm.3695 25344740

[220] Gao B Basic liver immunology. Cell Mol Immunol (2016) 13(3):265–6. 10.1038/cmi.2016.9 27041634 PMC4856812

[221] Robinson MW Harmon C O’Farrelly C. Liver immunology and its role in inflammation and homeostasis. Cell Mol Immunol (2016) 13(3):267–76. 10.1038/cmi.2016.3 27063467 PMC4856809

[222] Wisse E Vantnoordende JM Vandermeulen J Daems WT Pit cell – description of a new type of cell occurring in rat-liver sinusoids and peripheral-blood. Cell Tissue Res (1976) 173(4):423–35. 10.1007/BF00224305 991252

[223] Racanelli V Rehermann B. The liver as an immunological organ. Hepatology (2006) 43(2):S54–62. 10.1002/hep.21060 16447271

[224] Burt BM Plitas G Zhao ZG Bamboat ZM Nguyen HM Dupont B The lytic potential of human liver NK cells is restricted by their limited expression of inhibitory killer Ig-like receptors. J Immunol (2009) 183(3):1789–96. 10.4049/jimmunol.0900541 19587011 PMC3253491

[225] Gao B Jeong WI Tian ZG. Liver: an organ with predominant innate immunity. Hepatology (2008) 47(2):729–36. 10.1002/hep.22034 18167066

[226] Fiorentino DF Bond MW Mosmann TR 2 types of mouse T-helper cell 0.4. Th2 clones secrete a factor that inhibits cytokine production by Th1 clones. J Exp Med (1989) 170(6):2081–95. 10.1084/jem.170.6.2081 2531194 PMC2189521

[227] Brightbill HD Plevy SE Modlin RL Smale ST. A prominent role for Sp1 during lipopolysaccharide-mediated induction of the IL-10 promoter in macrophages. J Immunol (2000) 164(4):1940–51. 10.4049/jimmunol.164.4.1940 10657644

[228] Powell MJ Thompson SAJ Tone Y Waldmann H Tone M. Posttranscriptional regulation of IL-10 gene expression through sequences in the 3’-untranslated region. J Immunol (2000) 165(1):292–6. 10.4049/jimmunol.165.1.292 10861064

[229] Tan JC Indelicato SR Narula SK Zavodny PJ Chou CC. Characterization of interleukin-10 receptors on human and mouse cells. J Biol Chem (1993) 268(28):21053–9. 8407942

[230] Jinushi M Takehara T Tatsumi T Kanto T Miyagi T Suzuki T Negative regulation of NK cell activities by inhibitory receptor CD94/NKG2A leads to altered NK cell-induced modulation of dendritic cell functions in chronic hepatitis C virus infection. J Immunol (2004) 173(10):6072–81. 10.4049/jimmunol.173.10.6072 15528343

[231] Jinushi M Takehara T Tatsumi T Yamaguchi S Sakamori R Hiramatsu N Natural killer cell and hepatic cell interaction via NKG2A leads to dendritic cell-mediated induction of CD4(+) CD25(+) T cells with PD-1-dependent regulatory activities. Immunology (2007) 120(1):73–82. 10.1111/j.1365-2567.2006.02479.x 17052247 PMC2265878

[232] Travis MA Sheppard D TGF-beta activation and function in immunity. Ann Rev Immunol (2014) 32:51–82. 10.1146/annurev-immunol-032713-120257 24313777 PMC4010192

[233] Li MO Wan YY Sanjabi S Robertson AKL Flavell RA. Transforming growth factor-beta regulation of immune responses. Annu Rev Immunol (2006) 24:99–146. 10.1146/annurev.immunol.24.021605.090737 16551245

[234] Kang JS Liu C Derynck R. New regulatory mechanisms of TGF-beta receptor function. Trends Cell Biol (2009) 19(8):385–94. 10.1016/j.tcb.2009.05.008 19648010

[235] Weiss A Attisano L The TGFbeta superfamily signaling pathway. Wiley Interdiscip Rev Dev Biol (2013) 2(1):47–63. 10.1002/wdev.86 23799630

[236] Yang Y Han Q Hou Z Zhang C Tian Z Zhang J Exosomes mediate hepatitis B virus (HBV) transmission and NK-cell dysfunction. Cell Mol Immunol (2017) 14(5):465–75. 10.1038/cmi.2016.24 27238466 PMC5423088

[237] Zhang QF Yin WW Xia Y Yi YY He QF Wang X Liver-infiltrating CD11b-CD27- NK subsets account for NK-cell dysfunction in patients with hepatocellular carcinoma and are associated with tumor progression. Cell Mol Immunol (2016). 10.1038/cmi.2016.28 PMC5649104 27321064

[238] Yu J Wei M Becknell B Trotta R Liu S Boyd Z Pro- and antiinflammatory cytokine signaling: reciprocal antagonism regulates interferon-gamma production by human natural killer cells. Immunity (2006) 24(5):575–90. 10.1016/j.immuni.2006.03.016 16713975

[239] Sun C Fu BQ Gao YF Liao XF Sun R Tian ZG TGF-beta 1 down-regulation of NKG2D/DAP10 and 2B4/SAP expression on human NK cells contributes to HBV persistence. PLoS Pathog (2012) 8(3):e1002594 10.1371/journal.ppat.1002594 22438812 PMC3305436

[240] Sun C Sun H Zhang C Tian Z. NK cell receptor imbalance and NK cell dysfunction in HBV infection and hepatocellular carcinoma. Cell Mol Immunol (2015) 12(3):292–302. 10.1038/cmi.2014.91 25308752 PMC4654321

[241] Xu D Han Q Hou Z Zhang C Zhang J. miR-146a negatively regulates NK cell functions via STAT1 signaling. Cell Mol Immunol (2016). 10.1038/cmi.2015.113 26996068 PMC5549603

[242] Donatelli SS Zhou JM Gilvary DL Eksioglu EA Chen XH Cress WD TGF-beta-inducible microRNA-183 silences tumor-associated natural killer cells. Proc Natl Acad Sci U S A (2014) 111(11):4203–8. 10.1073/pnas.1319269111 24586048 PMC3964044

[243] Castriconi R Cantoni C Della Chiesa M Vitale M Marcenaro E Conte R Transforming growth factor beta 1 inhibits expression of NKp30 and NKG2D receptors: consequences for the NK-mediated killing of dendritic cells. Proc Natl Acad Sci U S A (2003) 100(7):4120–5. 10.1073/pnas.0730604100 12646700 PMC153058

[244] Koopman LA Kopcow HD Rybalov B Boyson JL Orange JS Schatz F Human decidual natural killer cells are a unique NK cell subset with immunomodulatory potential. J Exp Med (2003) 198(8):1201–12. 10.1084/jem.20030305 14568979 PMC2194228

[245] Tao Y Li YH Piao HL Zhou WJ Zhang D Fu Q CD56(bright) CD25(+) NK cells are preferentially recruited to the maternal/fetal interface in early human pregnancy. Cell Mol Immunol (2015) 12(1):77–86. 10.1038/cmi.2014.26 24793405 PMC4654367

[246] Sargent IL Borzychowski AM Redman CWG NK cells and human pregnancy – an inflammatory view. Trends Immunol (2006) 27(9):399–404. 10.1016/j.it.2006.06.009 16843067

[247] Li YH Zhou WH Tao Y Wang SC Jiang YL Zhang D The galectin-9/Tim-3 pathway is involved in the regulation of NK cell function at the maternal-fetal interface in early pregnancy. Cell Mol Immunol (2016) 13(1):73–81. 10.1038/cmi.2014.126 25578313 PMC4711677

[248] Higuma-Myojo S Sasaki Y Miyazaki S Sakai M Siozaki A Miwa N Cytokine profile of natural killer cells in early human pregnancy. Am J Reprod Immunol (2005) 54(1):21–9. 10.1111/j.1600-0897.2005.00279.x 15948769

[249] Zhang JH Chen ZL Smith GN Croy BA Natural killer cell-triggered vascular transformation: maternal care before birth? Cell Mol Immunol (2011) 8(1):1–11. 10.1038/cmi.2010.38 20711229 PMC3079746

[250] Ashkar AA Di Santo JP Croy BA. Interferon gamma contributes to initiation of uterine vascular modification, decidual integrity, and uterine natural killer cell maturation during normal murine pregnancy. J Exp Med (2000) 192(2):259–69. 10.1084/jem.192.2.259 10899912 PMC2193246

[251] Karimi K Arck PC. Natural killer cells: keepers of pregnancy in the turnstile of the environment. Brain Behav Immun (2010) 24(3):339–47. 10.1016/j.bbi.2009.09.015 19800965

[252] Aluvihare VR Kallikourdis M Betz AG. Regulatory T cells mediate maternal tolerance to the fetus. Nat Immunol (2004) 5(3):266–71. 10.1038/ni1037 14758358

[253] Zhu XY Zhou YH Wang MY Jin LP Yuan MM Li DJ Blockade of CD86 signaling facilitates a Th2 bias at the maternal-fetal interface and expands peripheral CD4(+)CD25(+) regulatory T cells to rescue abortion-prone fetuses. Biol Reprod (2005) 72(2):338–45. 10.1095/biolreprod.104.034108 15456701

[254] Guleria I Khosroshahi A Ansari MJ Habicht A Azuma M Yagita H A critical role for the programmed death ligand 1 in fetomaternal tolerance. J Exp Med (2005) 202(2):231–7. 10.1084/jem.20050019 16027236 PMC2213002

[255] Fu B Li X Sun R Tong X Ling B Tian Z Natural killer cells promote immune tolerance by regulating inflammatory T(H)17 cells at the human maternal-fetal interface. Proc Natl Acad Sci U S A (2013) 110(3):E231–40. 10.1073/pnas.1206322110 23271808 PMC3549088

[256] Vacca P Cantoni C Vitale M Prato C Canegallo F Fenoglio D Crosstalk between decidual NK and CD14(+) myelomonocytic cells results in induction of Tregs and immunosuppression. Proc Natl Acad Sci U S A (2010) 107(26):11918–23. 10.1073/pnas.1001749107 20547831 PMC2900704

[257] Zhang J Dunk CE Kwan M Jones RL Harris LK Keating S Human dNK cell function is differentially regulated by extrinsic cellular engagement and intrinsic activating receptors in first and second trimester pregnancy. Cell Mol Immunol (2017) 14(2):203–13. 10.1038/cmi.2015.66 26277900 PMC5301153

[258] Zhang N Bevan MJ Transforming growth factor-beta signaling controls the formation and maintenance of gut-resident memory T cells by regulating migration and retention. Immunity (2013) 39(4):687–96. 10.1016/j.immuni.2013.08.019 24076049 PMC3805703

[259] Ruggeri L Capanni M Urbani E Perruccio K Shlomchik WD Tosti A Effectiveness of donor natural killer cell alloreactivity in mismatched hematopoietic transplants. Science (2002) 295(5562):2097–100. 10.1126/science.1068440 11896281

[260] Miller JS Soignier Y Panoskaltsis-Mortari A McNearney SA Yun GH Fautsch SK Successful adoptive transfer and in vivo expansion of human haploidentical NK cells in patients with cancer. Blood (2005) 105(8):3051–7. 10.1182/blood-2004-07-2974 15632206

[261] Luevano M Madrigal A Saudemont A. Generation of natural killer cells from hematopoietic stem cells in vitro for immunotherapy. Cell Mol Immunol (2012) 9(4):310–20. 10.1038/cmi.2012.17 22705914 PMC4012863

[262] Cheng M Chen YY Xiao WH Sun R Tian ZG NK cell-based immunotherapy for malignant diseases. Cell Mol Immunol (2013) 10(3):230–52. 10.1038/cmi.2013.10 23604045 PMC4076738

[263] Berg M Lundqvist A McCoy P Samsel L Fan Y Tawab A Clinical-grade ex vivo-expanded human natural killer cells up-regulate activating receptors and death receptor ligands and have enhanced cytolytic activity against tumor cells. Cytotherapy (2009) 11(3):341–55. 10.1080/14653240902807034 19308771 PMC2736058

[264] Fujisaki H Kakuda H Shimasaki N Imai C Ma J Lockey T Expansion of highly cytotoxic human natural killer cells for cancer cell therapy. Cancer Res (2009) 69(9):4010–7. 10.1158/0008-5472.CAN-08-3712 19383914 PMC2716664

[265] Gong W Xiao W Hu M Weng X Qian L Pan X Ex vivo expansion of natural killer cells with high cytotoxicity by K562 cells modified to co-express major histocompatibility complex class I chain-related protein A, 4-1BB ligand, and interleukin-15. Tissue Antigens (2010) 76(6):467–75. 10.1111/j.1399-0039.2010.01535.x 20670353

[266] Melero I Johnston JV Shufford WW Mittler RS Chen LP. NK1.1 cells express 4-1BB (CDw137) costimulatory molecule and are required for tumor immunity elicited by anti-4-1BB monoclonal antibodies. Cell Immunol (1998) 190(2):167–72. 10.1006/cimm.1998.1396 9878117

[267] Imai C Iwamoto S Campana D. Genetic modification of primary natural killer cells overcomes inhibitory signals and induces specific killing of leukemic cells. Blood (2005) 106(1):376–83. 10.1182/blood-2004-12-4797 15755898 PMC1895123

[268] Fujisaki H Kakuda H Imai C Mullighan CG Campana D Replicative potential of human natural killer cells. Br J Haematol (2009) 145(5):606–13. 10.1111/j.1365-2141.2009.07667.x 19344420 PMC2776622

[269] Lapteva N Durett AG Sun JL Rollins LA Huye LL Fang J Large-scale ex vivo expansion and characterization of natural killer cells for clinical applications. Cytotherapy (2012) 14(9):1131–43. 10.3109/14653249.2012.700767 22900959 PMC4787300

[270] Ayello J Hochberg J Flower A Chu YY Baxi LV Quish W Genetically re-engineered K562 cells significantly expand and functionally activate cord blood natural killer cells: potential for adoptive cellular immunotherapy. Exp Hematol (2017) 46:38–47. 10.1016/j.exphem.2016.10.003 27765614

[271] Zhang H Cui YZ Voong N Sabatino M Stroncek DF Morisot S Activating signals dominate inhibitory signals in CD137L/IL-15 activated natural killer cells. J Immunother (2011) 34(2):187–95. 10.1097/CJI.0b013e31820d2a21 21304401 PMC3128544

[272] Shah NN Baird K Delbrook CP Fleisher TA Kohler ME Rampertaap S Acute GVHD in patients receiving IL-15/4-1BBL activated NK cells following T-cell-depleted stem cell transplantation. Blood (2015) 125(5):784–92. 10.1182/blood-2014-07-592881 25452614 PMC4311226

[273] Denman CJ Senyukov VV Somanchi SS Phatarpekar PV Kopp LM Johnson JL Membrane-bound IL-21 promotes sustained ex vivo proliferation of human natural killer cells. PLoS One (2012) 7(1):e30264. 10.1371/journal.pone.0030264 22279576 PMC3261192

[274] Siegler U Meyer-Monard S Jorger S Stern M Tichelli A Gratwohl A Good manufacturing practice-compliant cell sorting and large-scale expansion of single KIR-positive alloreactive human natural killer cells for multiple infusions to leukemia patients. Cytotherapy (2010) 12(6):750–63. 10.3109/14653241003786155 20491532

[275] Carlens S Gilljam M Chambers BJ Aschan J Guven H Ljunggren HG A new method for in vitro expansion of cytotoxic human CD3(-)CD56(+) natural killer cells. Hum Immunol (2001) 62(10):1092–8. 10.1016/S0198-8859(01)00313-5 11600215

[276] Morris RJ Chong LK Wilkinson GWG Wang ECY. A high-efficiency system of natural killer cell cloning. J Immunol Methods (2005) 307(1–2):24–33. 10.1016/j.jim.2005.08.015 16271362 PMC2843082

[277] Alici E Sutlu T Bojrkstrand B Gilljam M Stellan B Nahi H Autologous antitumor activity by NK cells expanded from myeloma patients using GMP-compliant components. Blood (2008) 111(6):3155–62. 10.1182/blood-2007-09-110312 18192509

[278] Lotzova E Savary CA Champlin RE Genesis of human oncolytic natural-killer-cells from primitive Cd34+Cd33- bone-marrow progenitors. J Immunol (1993) 150(12):5263–9. 7685792

[279] Giarratana MC Verge V Schmitt C Bertho JM Kobari L Barret C Presence of primitive lymphoid progenitors with NK or B potential in ex vivo expanded bone marrow cell cultures. Exp Hematol (2000) 28(1):46–54. 10.1016/S0301-472x(99)00131-9 10658676

[280] Yu Y Hagihara M Ando K Gansuvd B Matsuzawa H Tsuchiya T Enhancement of human cord blood CD34(+) cell-derived NK cell cytotoxicity by dendritic cells. J Immunol (2001) 166(3):1590–600. 10.4049/jimmunol.166.3.1590 11160200

[281] Kalberer CP Siegler U Wodnar-Filipowicz A Human NK cell development in NOD/SCID mice receiving grafts of cord blood CD34(+) cells. Blood (2003) 102(1):127–35. 10.1182/blood-2002-07-2024 12637322

[282] Perez SA Sotiropoulou PA Gkika DG Mahaira LG Niarchos DK Gritzapis AD A novel myeloid-like NK cell progenitor in human umbilical cord blood. Blood (2003) 101(9):3444–50. 10.1182/blood-2002-05-1501 12506032

[283] Spanholtz J Tordoir M Eissens D Preijers F van der Meer A Joosten I High log-scale expansion of functional human natural killer cells from umbilical cord blood CD34-positive cells for adoptive cancer immunotherapy. PLoS One (2010) 5(2):e9221. 10.1371/journal.pone.0009221 20169160 PMC2821405

[284] Spanholtz J Preijers F Tordoir M Trilsbeek C Paardekooper J de Witte T Clinical-grade generation of active NK cells from cord blood hematopoietic progenitor cells for immunotherapy using a closed-system culture process. PLoS One (2011) 6(6):e20740 10.1371/journal.pone.0020740 21698239 PMC3116834

[285] Vitale C Cottalasso F Montaldo E Moretta L Mingari MC Methylprednisolone induces preferential and rapid differentiation of CD34(+) cord blood precursors toward NK cells. Int Immunol (2008) 20(4):565–75. 10.1093/intimm/dxn014 18310065

[286] Kim PS Kwilas AR Xu W Alter S Jeng EK Wong HC IL-15 superagonist/IL-15RalphaSushi-Fc fusion complex (IL-15SA/IL-15RalphaSu-Fc; ALT-803) markedly enhances specific subpopulations of NK and memory CD8+ T cells, and mediates potent anti-tumor activity against murine breast and colon carcinomas. Oncotarget (2016) 7(13):16130–45. 10.18632/oncotarget.7470 26910920 PMC4941302

[287] Glienke W Esser R Priesner C Suerth JD Schambach A Wels WS Advantages and applications of CAR-expressing natural killer cells. Front Pharmacol (2015) 6:21. 10.3389/Fphar.2015.00021 25729364 PMC4325659

[288] Sahm C Schonfeld K Wels WS. Expression of IL-15 in NK cells results in rapid enrichment and selective cytotoxicity of gene-modified effectors that carry a tumor-specific antigen receptor. Cancer Immunol Immunother (2012) 61(9):1451–61. 10.1007/s00262-012-1212-x 22310931 PMC11029748

[289] Benson DM Jr Bakan CE Zhang S Collins SM Liang J Srivastava S IPH2101, a novel anti-inhibitory KIR antibody, and lenalidomide combine to enhance the natural killer cell versus multiple myeloma effect. Blood (2011) 118(24):6387–91. 10.1182/blood-2011-06-360255 22031859 PMC3490103

[290] Ruggeri L Urbani E Andre P Mancusi A Tosti A Topini F Effects of anti-NKG2A antibody administration on leukemia and normal hematopoietic cells. Haematologica (2016) 101(5):626–33. 10.3324/haematol.2015.135301 26721894 PMC5004363

[291] Chang YH Connolly J Shimasaki N Mimura K Kono K Campana D. A chimeric receptor with NKG2D specificity enhances natural killer cell activation and killing of tumor cells. Cancer Res (2013) 73(6):1777–86. 10.1158/0008-5472.CAN-12-3558 23302231

[292] Tay SS Carol H Biro M. TriKEs and BiKEs join CARs on the cancer immunotherapy highway. Hum Vaccin Immunother (2016) 12(11):2790–6. 10.1080/21645515.2016.1198455 27322989 PMC5137511

[293] Schmohl JU Felices M Oh F Lenvik AJ Lebeau AM Panyam J Engineering of anti-CD133 trispecific molecule capable of inducing NK expansion and driving antibody-dependent cell-mediated cytotoxicity. Cancer Res Treat (2017). 10.4143/crt.2016.491 28231426 PMC5654165

